# Liposome-based responsive MRI contrast agents

**DOI:** 10.1039/d6cc05928g

**Published:** 2026-10-09

**Authors:** Anna M. Duncan, Connor M. Ellis, James P. Smith, Jason J. Davis

**Affiliations:** a Department of Chemistry, University of Oxford South Parks Road Oxford OX1 3QZ UK Jason.davis@chem.ox.ac.uk +44 01865 275 914 +44 01865 275 914

## Abstract

Liposomes present a promising architecture for the design of responsive MRI CAs. The ability to co-encapsulate diagnostic and therapeutic payloads further allows for (targeted) imaging and treatment of disease *in vivo*. Tuneable control over embedded motifs, or modification of the bilayer phospholipids themselves, can support a programmable response to disease microenvironments. In this review we summarise the design and underpinning theoretical principles relevant to the production of next-generation diagnostically potent responsive liposome-based MRI CAs.

## Abstract 2

Stimuli-responsive MRI contrast agents based on liposomal scaffolds are promising architectures for future clinical utilisation. Herein we explore how the highly tuneable bilayer membrane facilitates engineered modulation in relaxometric properties.

## Graphical abstract

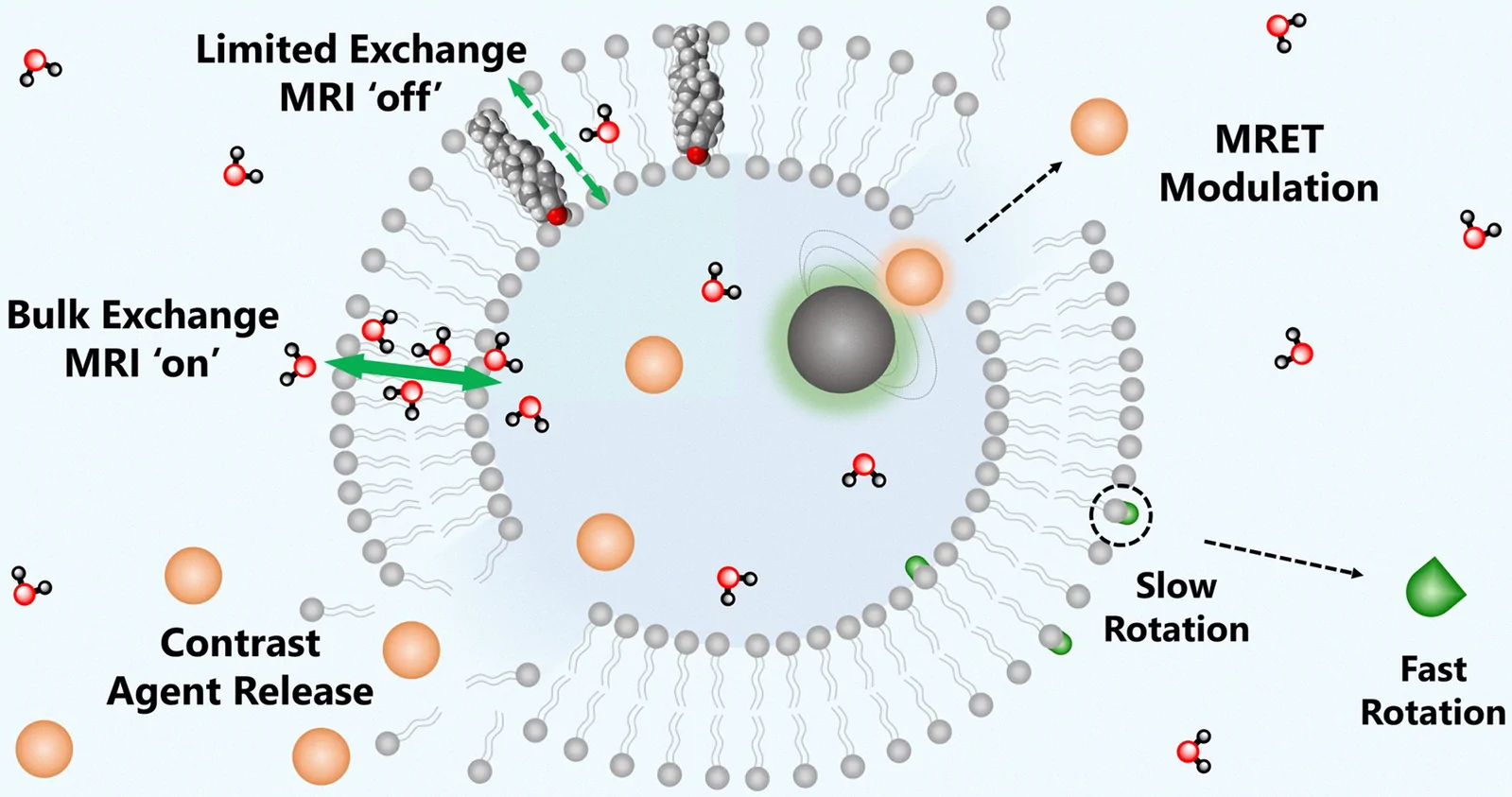

## 1. Introduction

Liposomes are a class of synthetic vesicles designed to encapsulate (bio)materials within an amphiphilic biomimetic membrane, shielding internal cargo from undesired/off-target interactions, improving biocompatibility.^1^ These membranes can be composed of phospholipids, polymers, or specific phospholipid–polymer hybrids, synthetically engineered to be similar to cells in terms of compartmentalisation and an impeded cross-membrane passage of highly polar or charged moieties.^2–4^ Liposome scaffolds can be tailored to the intended *in vivo* application through the careful selection of optimised lipid ratios, and incorporated therapeutic and/or diagnostic agents.^5^ A rational and tuneable design can be leveraged to achieve stimuli responsivity, controlled release, and favourable *in vivo* biocompatibility profiles.^6^ Internal cargo such as hydrophilic drugs can be added at the point of lipid hydration,^7^ and has included anticancer Pt-based agents (carboplatin,^8^ cisplatin^9^), nucleoside formulations (cytarabine),^10^ or anthracycline-based systems (doxorubicin,^11^ daunorubicin^12^). Due to their tuneability and immunogenicity, these constructs are involved in a range of clinical applications,^13^ including recently as an adjuvant in SARS–CoV-2 vaccines. In the latter application, encapsulation of mRNA within the inner aqueous core enables cargo protection long enough *in vivo* to produce the spike protein^14^ and thereby elicit a successful immune response.^15^ To date, some 15 liposomal nanomedicines have been approved by the U.S. Food and Drug Administration (FDA), possessing both improved targeting and reduced off-target toxicity when compared to their non-encapsulated counterparts.^16,17^ In particular, the encapsulation of metallo-^18^ and organic drugs (Doxil®, DaunoXome® and Depocyt®),^19^ for cancer therapy. Doxil®, used in the treatment of ovarian and breast cancer, possesses a poly(ethylene glycol) modified (PEGylated), cholesterol modified liquid-phase liposomal membrane promoting both prolonged drug circulation and a high internal loading of doxorubicin.^17^ In comparison to Doxil®, Depocyt® is approved to treat lymphomatous meningitis, and DaunoXome® for the treatment of paediatric brain tumours.^20,21^ Encapsulated cisplatin (Lipoplatin^TM^) has successfully completed Phase III clinical trials, demonstrating an ever-growing research interest in liposomal formulations.^21,22^ Compared with cisplatin, Lipoplatin^TM^ offers a ∼20-fold longer half-life (117 h *vs.* 6 h, enabling maximised cancer cell damage), and improved tolerability and reduced nephrotoxicity even after repeated doses.^23^

It is, therefore, natural to draw upon established clinical liposome formulations to inform and advance the design and performance of these and closely related architectures as clinical imaging agents.^24^ Extensive research into paramagnetic liposomes has, for example, sought to validate their use in visualising pathophysiological processes by magnetic resonance imaging (MRI),^25^ complementing their established therapeutic applications.^26^ With these, effective contrast generation is achieved by selection of an appropriate (super-)paramagnetic dopant within the aqueous internal space (*e.g.*, Gd(iii)- or Mn(ii)-doped nanoparticles/chelates, or iron oxide nanoparticles).^27^ Co-encapsulation of imaging agents alongside therapeutics, natively supports the development of theranostic liposomes (see Fig. 1).^28^ Within the context of clinical diagnostics, MRI is a dominant non-invasive imaging tool, offering superior spatial resolution and tissue contrast when compared to other clinical imaging modalities (positron emission tomography (PET), optical imaging (OI) and single-photon emission computed tomography (SPECT)).^29^ Adequate distinction between tissues can be obtained in the absence of MRI contrast agents (CAs), but information density, and hence diagnostic accuracy, can be dramatically improved through their use.^30^

**Fig. 1:**
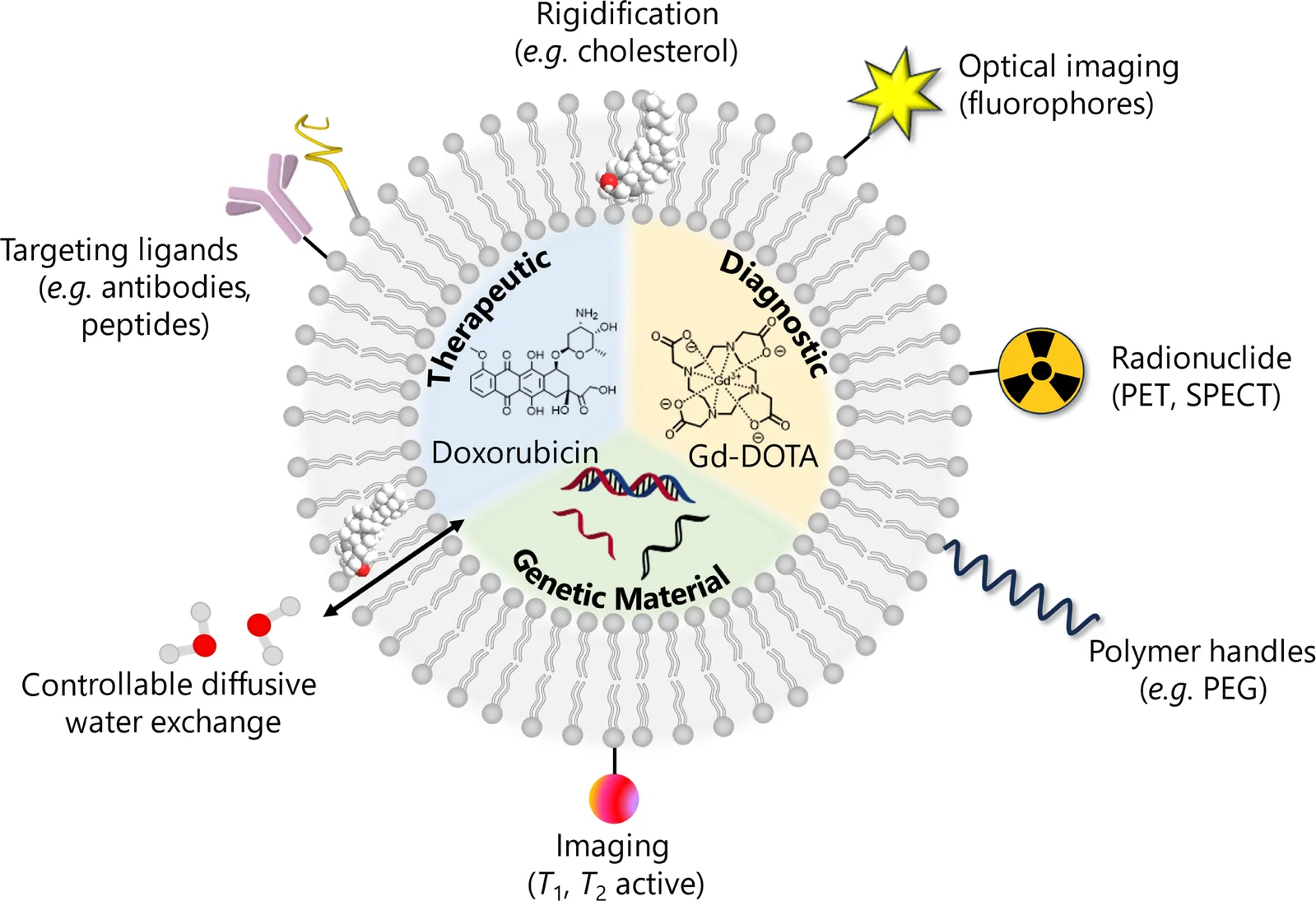
Liposomal scaffolds enable internal cargo encapsulation or surface modification with diagnostic and/or therapeutic motifs. Incorporation of tuneable levels of cholesterol provides a handle over which diffusive water exchange between bulk and the internal water pool can be controlled. Diagnostic cargo may include *T*_1_ or *T*_2_ active chelates/nanoparticles for MRI applications, fluorophores for optical imaging, or radionuclides for PET (positron emission tomography)/SPECT (single photon emission computed tomography) imaging. PEGylation improves biocompatibility by providing a steric barrier to protein adsorption, reducing rapid immune system clearance, whilst active targeting ligands such as antibodies or peptides enable selective targeting of disease sites or specific regions of interest (ROIs).

In this review we illustrate intrinsic design properties that define liposome CA performance, and demonstrate how these features can be further manipulated to promote environmentally-responsive imaging. The latter is specifically achieved through modulation of one (or more) of the intrinsic Solomon–Bloembergen–Morgan parameters that govern overall relaxivity.^31^ We additionally explore a range of image-supporting payloads, alongside the influence of liposome size, composition, and membrane properties on contrast generation and responsiveness. By connecting liposome design principles with the mechanisms underlying paramagnetic MRI contrast generation, we present design considerations relevant to the development and clinical translation of a new generation of stimuli-responsive contrast agents.

## 2. MRI theory

### 2.1. General MRI theory

MRI CAs are classified by the contrast they generate, either *T*_1_ or *T*_2_, and can be further divided into nanoparticulate or molecular CAs.^32^*T*_1_ agents (typically Gd(iii), Mn(ii)-based) enhance the rate of longitudinal (spin–lattice) relaxation, brightening the image. Historically, clinically employed *T*_1_ contrast agents are molecular, typically gadolinium-based (GBCAs), including macrocyclic chelates such as Dotarem® (Gd–DOTA), Gadoteridol (Gd–HP–DO3A), and linear ionic chelates Primovist® (Gd–EOB–DTPA) or Magnevist® (Gd–DTPA).^33^ Macrocyclic groups offer significantly diminished %Gd(iii) release (<0.1%) compared with both non-ionic/ionic linear chelating scaffolds (1.9% over 15 days for Magnevist®), arising from an increased thermodynamic stability coefficient.^34^*T*_2_ agents (commonly superparamagnetic iron oxide nanoparticle, SPION, based) have the analogous effect on transverse (spin–spin) relaxation processes, darkening the image. SPIONs induce strong local magnetic inhomogeneities causing a loss of spin coherence/rapid dephasing of nearby ^1^H nuclei.^35^ Both classes of contrast can be readily integrated within liposomal architectures.^36^

In an MRI scanner, initially, the protons are aligned with the externally applied magnetic field (*B*_0_), with the net magnetisation vector (*M*_*z*_) aligned with the *z*-axis.^37^ For *T*_1_ acquisition, the net magnetisation vector is inverted by application (and subsequent removal) of a 180° radiofrequency (RF) pulse.^38^ A subsequent spin–lattice relaxation process enables the perturbed spins to return to equilibrium (aligning with *B*_0_). After time *t* (inversion time), a 90° RF pulse is applied to modulate the magnetisation onto the *xy*-plane to enable detection of the MR signal. Comparatively, for *T*_2_ acquisition, a 90° RF pulse causes the net magnetisation vector to lie along the *xy*-plane (*M*_*xy*_) before spins lose coherence (primarily through a spin–spin relaxation process), and return to equilibrium (*M*_*xy*_ = 0).^39^ Contrast agent potency is typically described by its relaxivity (*r*_1_ or *r*_2_), defined by the slope of 1/*T*_*i*_ (where *i* = 1 or 2) against the concentration of paramagnetic agent (*e.g.*, [Gd(iii)]).^40^ Traditional *T*_1_ contrast agents possess moderate relaxivity values, for example, Magnevist® yields a *r*_1_ = 3.2 mM^−1^ s^−1^ and *r*_2_ = 5.1 mM^−1^ s^−1^, with Primovist® displaying a *r*_1_ = 5.3 mM^−1^ s^−1^ and *r*_2_ = 7.2 mM^−1^ s^−1^ (at 298 K, 3 T).^41,42^ In obtaining a greater insight into contrast agent application, the relaxometric ratio (*i.e.*, *r*_2_/*r*_1_) is useful; within this, it is generally accepted that values of *r*_2_/*r*_1_ < 5 are indicative of *T*_1_-weighted MRI CAs, whilst > 8 denotes suitability for *T*_2_-weighted image acquisition.^43^

Paramagnetic CAs are able to interact with water molecules through three distinct mechanisms, namely inner-sphere (IS), second-sphere (SS) or outer-sphere (OS) relaxation (the latter explained by a Hwang–Freed framework),^44^ before diffusing back into bulk.^45^ The degree by which these three spheres contribute to relaxivity is largely determined by the nature of the CA.^46^ Molecular chelates are typically limited to IS and SS effects, whereas nanoparticulate scaffolds, in particular, can promote significant OS contributions.^47^ The Solomon-Bloembergen-Morgan (SBM) equations provide a theoretical framework for assessing how these (or closely related) parameters govern overall relaxivity, an understanding of which underpins responsive contrast agent design and application.^47^

### 2.2. Engineering responsivity

Liposomes present an opportunity to influence interactions between CA and water, through both confinement effects (altered rotational dynamics, reduced interactions of paramagnetic agent with bulk water) and a uniquely tuneable hydrophobic barrier.^48^ The former is as a result of their well-defined hydrophilic internal cavity, which promotes a localised, concentrated volume of encapsulated material. Specifically, encapsulation of the paramagnetic cargo yields a high payload concentration of 10^4^–10^5^ molecular chelates per vesicle.^49^ Superparamagnetic dopants can also be incorporated, a consideration of which is included in Section 4.3.

The efficiency of paramagnetic CAs is, as noted above, strongly influenced by water accessibility. This can ultimately be quantified by the residence time (*τ*_M_) which describes the corresponding rate (*k*_ex_ = 1/*τ*_M_) at which water exchanges into/out of the IS (where it undergoes relaxation at the paramagnetic centre).^45^ Within a lipid architecture, IS exchange is impacted by the limited water pool, which diminishes the number of available bulk water molecules that can directly (and effectively) interact with Gd(iii) by an IS mechanism per unit time. This internally limiting water pool is inherently present when paramagnetic agents are confined within liposomal structures whereby cross-bilayer exchange is deliberately limited. By considering intrinsic and defining SBM correlation times, a mechanistic framework for the observed relaxivity suppression of encapsulated paramagnetic cargo can be developed, enabling a platform for retrospective restoration of image contrast in an environmentally-responsive manner (see Section 5).

To examine this further; it is known that the IS residence time of H_2_O coordinated to Gd–DOTA is 97 ns at 37 °C,^50^ allowing, in unrestricted bulk solution, a single Gd-centre to directly coordinate with a theoretical maximum of 10.3 million (distinct) water molecules per second (in the absence of diffusion limits).^51,52^ This drops significantly on bilayer encapsulation. For example, liposomal structures of 100 nm possess an internal volume of ∼500 000 nm^3^, which equates to approximately 20 million water molecules. When imaging with a 1.5 T clinical scanner this results in an MRI ‘silenced’/‘off’ state, as internalised Gd(iii) are able to relax all available water molecules (when [Gd] loading is high, *i.e.*, number of Gd : H_2_O molecules ∼ 1 : 1000) on a timescale that is short relative to the measurement time *t*. The limiting water pool within a liposome can therefore be approximated by a “proxy *τ*_M_”, which considers the average time taken for a chelate to coordinate with an unrelaxed water molecule. This provides a framework for evaluating the impact of changing the permeability/water flux rates across the lipid bilayer on overall relaxivity for core-doped paramagnetic agents, and how the potential magnitude of contrast enhancement (*r*_1_ switch) relates directly to how highly limiting/non-limiting the accessible water pool is in response to external stimuli (Fig. 2).

**Fig. 2:**
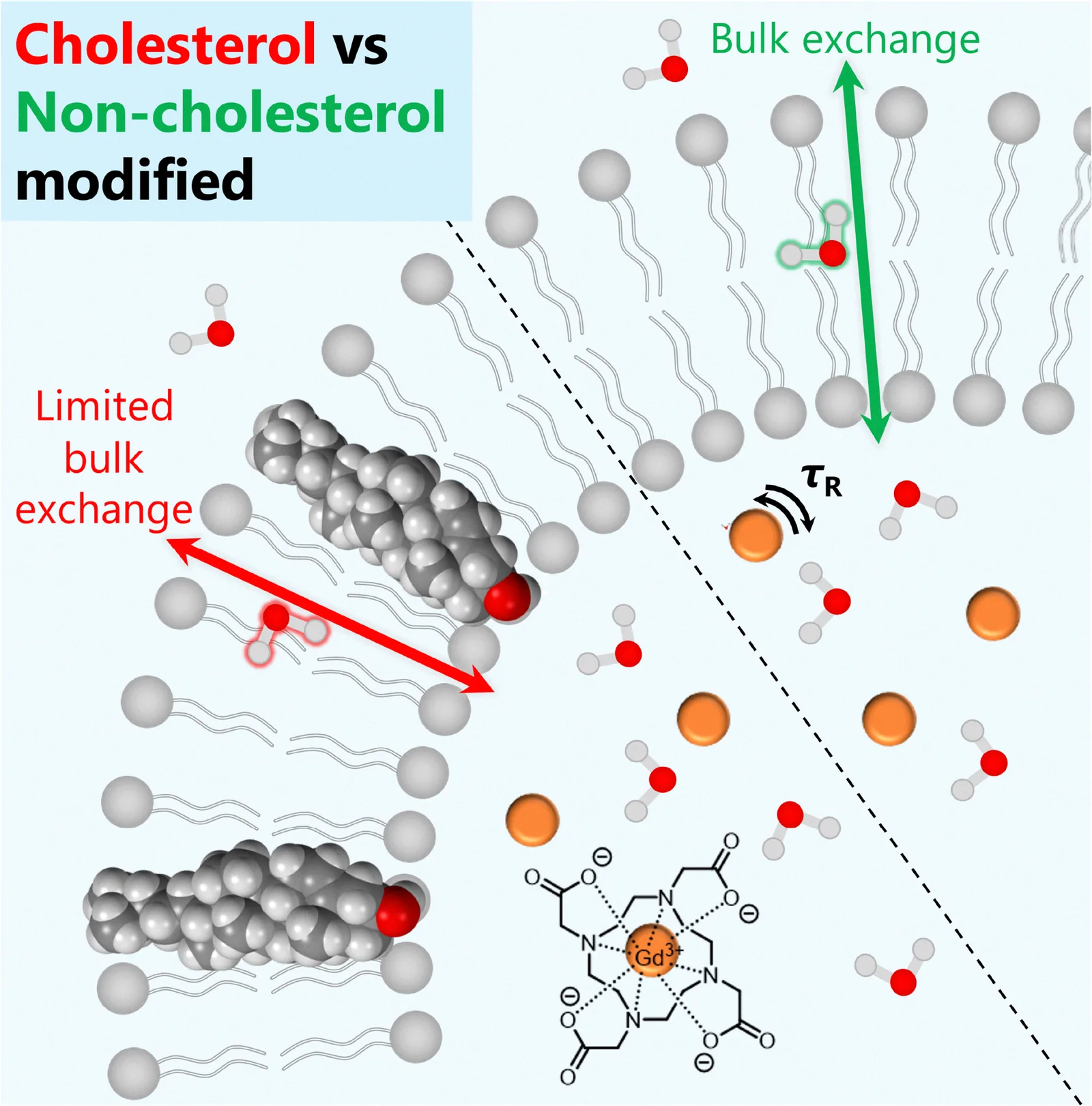
Schematic representation of Gd(iii) chelates encapsulated within the aqueous lumen of a liposome. The lipid bilayer acts as a diffusion barrier, limiting cross-membrane water exchange and diminishing relaxivity, which can be modelled by changing *τ*_M_ as a proxy. Cholesterol incorporation further silences MRI contrast by more highly restricting cross-bilayer diffusive water exchange. This results in a greater proportion of non-relaxivity inducing interactions between Gd(iii) chelates and water molecules within the intravesicular pool between successive RF pulses, reducing the efficiency of paramagnetic relaxation.

Further to the native hydrophobicity imposed by the central acyl chains within the lipid bilayer, the incorporation of cholesterol at levels representative of typical mammalian cells (30–40 mol%, defined w.r.t. moles of lipid)^53^ can further diminish the passive flux of water across the bilayer (Fig. 2).^54^ Cholesterol insertion between phospholipid molecules specifically increases bilayer packing density/order resulting in lower membrane permeability, generating a further barrier to cross-bilayer water exchange.^49^ It is known that, for example, as cholesterol content increases from 0–40 mol%, the water diffusive permeability decreases from 63 × 10^−4^ cm s^−1^ to 8.7 × 10^−4^ cm s^−1^, corresponding to a decrease in *r*_1_ by 1 mM^−1^ s^−1^.^49,55^ The introduced concept of an elongated “proxy *τ*_M_” can therefore be used to explain this reduction in *r*_1_ observed for highly cholesterol doped Gd–DOTA encapsulated liposomes compared with their non-cholesterol modified counterparts. Incorporation of cholesterol is not the only strategy by which MRI can be turned ‘off’ in paramagnetically doped liposomes. Section 3.3 highlights how relaxivity can be further tuned down by controlling the packing density of the native lipids, intrinsically linked to the degree of saturation within the phospholipid tails. When rapid cross-bilayer water flux is retrospectively established (for example through hydrolytic, or non-destructive approaches such as aquaporin integration, see Sections 5.1 and 5.2), an increase in *r*_1_ can be achieved. Furthermore, in cases where this activation of cross bilayer water exchange is deliberately restored in the presence of certain external microenvironmental stimuli, this enables a means by which disease status can be understood, providing diagnostic information relating to differences in local pH, temperature,^56^ or enzymatic presence (see Section 5).^57,58^

In comparison to liposome core-doped contrast agents, the relaxivity of peripheral bilayer-anchored moieties is elevated (*e.g.*, *r*_1_ ∼ 10 mM^−1^ s^−1^)^59^ compared to their core-confined paramagnetic analogues (∼1–2 mM^−1^ s^−1^),^60^ due to both an elongated *τ*_R_ and unrestricted bulk diffusive water access.^61,62^ In one such example of bilayer-doped paramagnetic modification, Hak *et al.* demonstrated that the lipid headgroup could be covalently tethered to Gd–DOTA to achieve an improved *r*_1_ = 11.8 mM^−1^ s^−1^ (298 K, 1.41 T) when compared to molecular Gd–DOTA (*r*_1_ ∼ 4.0 mM^−1^ s^−1^, 1.41 T).^63^ This substantially elevated *r*_1_ was rationalised through application of a Lipari–Szabo approach to SBM theory (where global and local rotational characteristics can be decoupled).^64,65^ In a further related example, Kielar *et al.* functionalised Gd–DOTA with two hydrophobic C_12_ alkyl chains to deliberately engineer greater lipophilicity, where membrane embedding (10 mol% Gd–chelate) resulted in a 135% increase in *r*_1_ (40.0 mM^−1^ s^−1^, 0.47 T, 298 K) compared with the singular C_12_ alkyl chain analogue (*r*_1_ = 17.0 mM^−1^ s^−1^, 0.47 T), owing to slowed rotational dynamics of the bis-alkyl analogue once in the lipid membrane.^42,66^ Of course, manipulation of either of these introduced correlation times (*τ*_R_, *τ*_M_), by (dis-)assembly or enhancing/restricting local water access, can be invoked to generate a potent environmentally-responsive relaxometric switch enabling an insight into the local microenvironment (see Section 5).

## 3. Liposome design features

An understanding of lipid design (composition, size, and relative component formulation), as utilised in approved drug delivery systems, of course provides a framework for the generation of derived MRI CAs. This includes a considered engineering of anti-fouling character, circulation lifetime, targeting, triggered aggregation and water access.^67,68^ This Section considers these key features in turn and discusses their direct relevance to responsive liposomal MRI CAs. Section 4 will elaborate on this to discuss the range of payloads, both chelate-and nanoparticle-based that can be incorporated within the chosen liposomal scaffold to generate (responsive) MRI contrast.

### 3.1. Tuning liposomal diameter

Clearance rates by the reticuloendothelial system (RES) are not only dependent on surface charge, but also diameter.^69^ Therefore, for *in vivo* applications, a size range of ∼50–200 nm is considered optimal for idealised circulation times in the bloodstream, as well as enabling a sufficient payload capacity.^70^ All 15 current FDA approved liposomal formulations possess controlled sizes within this range.^71^ Such control over diameter is easily achieved in liposomal structures through the selection of filter pore size during extrusion.^72^ As described in Section 2.2, for internally paramagnetically-doped liposomes, the ability to generate effective MRI contrast relies on the number of ‘bulk’ or ‘accessible’ water molecules surrounding the enclosed (super-)paramagnetic material. These, then, are most primed to be responsive should this change. Of course, the water volume/number of water molecules encapsulated correlates directly with size, with 400 nm liposomes possessing ∼500× more internal water molecules than their 50 nm equivalents. To this end, several studies have investigated how changing the hydrodynamic radius of liposomes affects their downstream properties including pharmacokinetics, payload trapping efficiency, and MRI CA performance.^60^ The number of water molecules that are able to interact with Gd(iii) is proportional to [Gd], and inversely proportional to the internal water pool volume. CA relaxation (*R*_1_), therefore, correlates with lipid bilayer permeability and surface area-to-volume (*S*/*V*) ratio. In one such example, Ghaghada *et al.* investigated *T*_1_ relaxivity at four different sizes (50, 100, 200 and 400 nm liposomes, comprised of 1,2-dipalmitoyl-*sn*-glycero-3-phosphocholine (DPPC) : cholesterol : 1,2-distearoyl-*sn*-glycero-3-phosphoethanolamine-*N*-[methoxy(polyethylene glycol)-2000] (DSPE-PEG2000) in a 55 : 40 : 5 molar ratio) and three [Gd] (200, 350 and 500 mM gadodiamide in the internal pool), reporting an inverse correlation between liposome size and *r*_1_.^73^ This was attributed to the *S*/*V* increasing as size decreases, increasing *r*_1_. Whilst potentially counterintuitive, the greater *S*/*V* ratio of smaller liposomes allows for relatively higher levels diffusive water access across the membrane into the internal cavity. This is attributed to the relative increase in total cross-bilayer water exchange as size decreases, enhancing observed *r*_1_. For liposomes above a critical encapsulated [Gd], supply of unrelaxed water molecules to paramagnetic centres becomes the limiting factor for measured relaxivity. In such conditions, the measured relaxivity is dominated by access to bulk water (and trans-bilayer water exchange rate), rather than the number of water molecules within the internal pool.

### 3.2. Lipid headgroup effects

Phospholipid headgroups may be zwitterionic, negative, or positively charged. Globally neutral (zwitterionic) headgroups are the most abundant in mammalian cell membranes due to their ‘antifouling’ nature. Antifouling refers to the prevention of nonspecific adsorption of proteins such as immunoglobulins (IgG) or fibrinogen, which would otherwise encourage *in vivo* recognition and subsequent uptake by macrophages and/or leukocytes.^74^ This reduces the likelihood of opsonisation *in vivo*, improving both circulation time and hence accumulation at the target site (*e.g.*, by the enhanced permeability and retention effect).^75^ Electrostatic interactions with serum proteins increase as the magnitude of nanoparticle surface charge becomes increasingly positive or negative, leading to more rapid clearance by the reticuloendothelial system.^76^ Therefore, globally neutral (*e.g.* zwitterionic) nanoparticle (NP) species are preferred for *in vivo* applications.^69^ Zwitterionic lipids are also relatively easy to formulate due to their low gel-to-liquid phase transition temperature.^77,78^

There are a wide variety of zwitterionic lipids based on phosphatidylcholine (PC) or phosphatidylethanolamine (PE) structures including 1-palmitoyl-2-oleoyl-*sn*-glycero-3-phosphocholine (POPC), 1,2-dioleoyl-*sn*-glycero-3-phosphoethanolamine (DOPE), dioleoyl phosphatidylcholine (DOPC), 1,2-distearoyl-*sn*-glycero-3-phosphoethanolamine (DSPE), DPPC, 1,2-distearoyl-*sn*-glycero-3-phosphocholine (DSPC) and 1,2-dipalmitoyl-*sn*-glycero-3-phosphoethanolamine (DPPE), shown in Fig. 3.^79^ These lipids can also be PEGylated (commonly with PEG-2000, *e.g.*, DSPE-PEG2000) and used in low molar percentage in combination with cholesterol and other standard lipids^80^ as a stabiliser and stealth agent to improve the *in vivo* performance of anticancer drugs.^81,82^ The DOPE head-group has been used within MRI CA applications to bear a Gd-bound chelating agent through covalent modification (DOPE–Gd–DOTA).^83^ DOPE–Gd–DOTA, as well as the analogous DSPE–Gd–DOTA, are both commercially available chelator lipids which have been used for the purposes of arterial, joint, tumour, liver and brain imaging.^84^ Such paramagnetic lipids when incorporated into liposomal structures have facilitated favourable *in vivo* half-lives and biodistribution, with maximum contrast typically reported within 1–2 h, and persisting up to 24 h, depending on the formulation employed.^83^ These MRI agents are known as blood-pool CAs, as they persist in the cardiovascular system, yielding ideal contrast for quantitative perfusion imaging.^85^ Alternatively, lipid head groups can be modified with maleimide (Mal) functionality to improve specific targeting, for example DSPE–PEG–Mal.^86^ Iyer *et al.* have, for example, reported that low molar percent incorporation of DSPE–PEG–maleimide into liposomal structures installs an active surface for the insertion of human single chain antibodies (scFvs) which are then anchored in the membrane of pre-formed liposomes.^87^ Straub *et al.* have also described MRI-based molecular imaging using maleimide-functionalised lipids, which facilitated active targeting of epicardium-derived stromal cells by conjugating to targeting peptides.^88^ Headgroup functionalisation within zwitterionic phospholipids, then, spans PEGylation (circulation), *T*_1_-active chelate tethering (imaging), and incorporation of reactive handles for covalent conjugation of targeting moieties such as antibodies or peptides (active targeting).

**Fig. 3:**
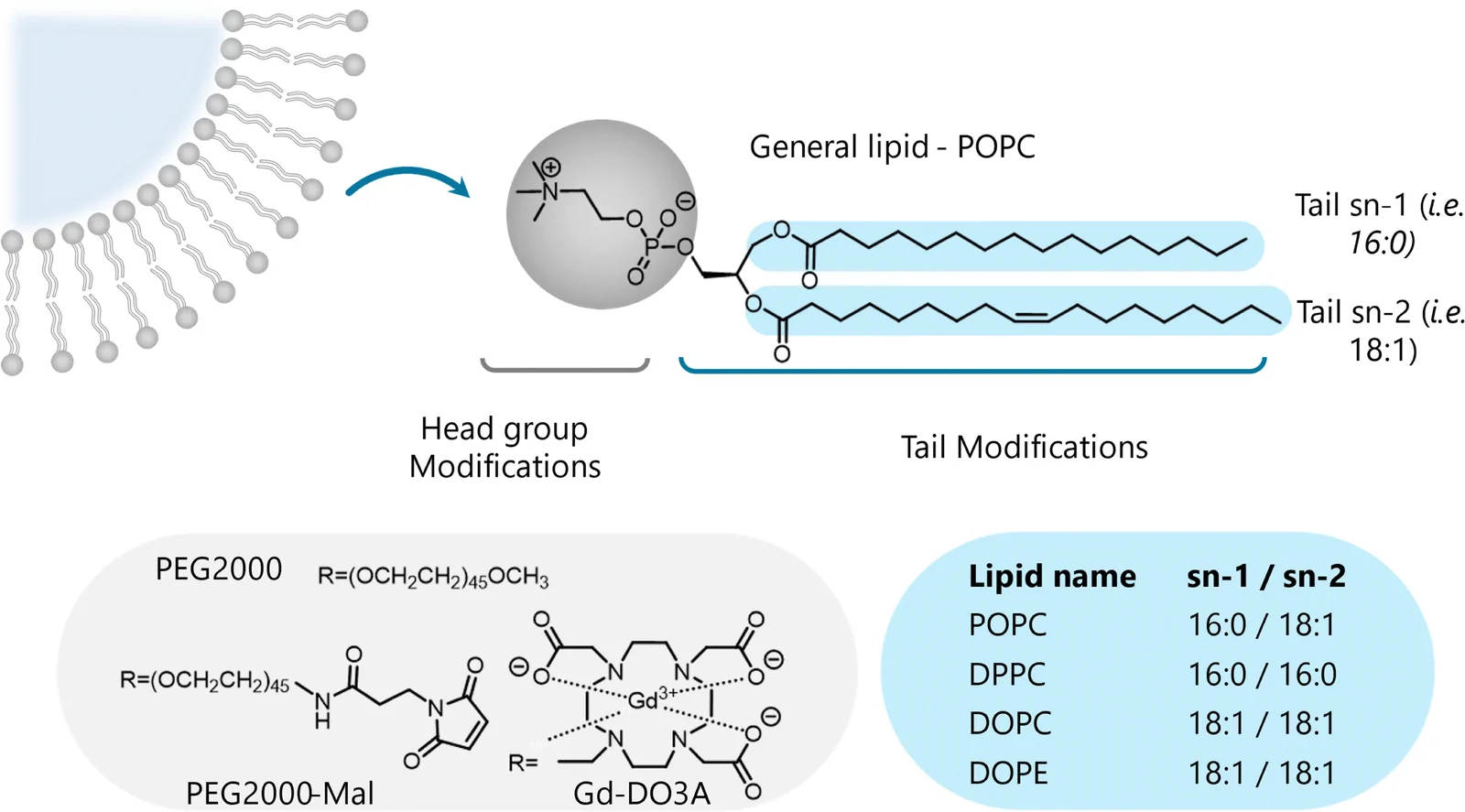
The constituent head or tail groups within phospholipids can be modulated to impart desired properties. For example, altering the level of unsaturation within the tails directly affects both the membrane packing density and bilayer fluidity/permeability. This dictates the rate of water flux across the bilayer and the extent by which bulk water can interact with encapsulated paramagnetic payloads and undergo relaxation. By functionalising the phospholipid head groups with paramagnetic chelates such as Gd–DO3A, this imparts a *T*_1_ imaging capability. Alternatively, PEG2000 modification (shown in head group modifications) has been used to extend circulation lifetime *in vivo*, improving the associated stability profile. When the PEG2000 moiety is further functionalised with maleimide, or biotin groups, this enables the facile covalent attachment of targeting moieties (*e.g.*, maleimide readily reacts with thiol-containing structures, biotin binds with high affinity to streptavidin). Sn-1 and sn-2 refer to the locations of the acyl chains; the sn-1 tail is positioned at the top of the glycerol backbone and is usually saturated (tight chain packing; 16 : 0 refers to the presence of 16 C atoms and no double bonds), whilst the sn-2 is generally unsaturated (kinked tail structure, adds fluidity to the membrane; 18 : 1 refers to the presence of 18 C atoms and a single double bond).

In comparison to zwitterionic lipids, cationic lipids are known for their ability to electrostatically interact with anionic molecules such as RNA or lipids, for facile cell uptake by endocytosis to reside within cell endosomes.^89,90^ Cationic lipids are also able to associate with NPs which possess a negative surface charge, such as iron oxide nanoparticles (IONPs), allowing facile bilayer encapsulation.^91^ Cationic lipids include 1,2-dioleoyl-3-trimethylammonium-propane (DOTAP), 1,2-di-O-octadecenyl-3-trimethylammonium propane (DOTMA), didodecyldimethylammonium bromide (DDAB) and o,o′-ditetradecanoyl-*N*-(α-trimethylammonioacetyl)diethanolamine chloride (DC-6-14).^92–94^ Mirroring the use of DOTAP in DNA transfection studies,^95^ Guenoun *et al.* have, for example, utilised DOTAP in combination with DPPC and cholesterol in a 20 : 33 : 47 molar ratio to encapsulate Gd–DTPA for the efficient labelling and tracking of mesenchymal stem cells.^96^ The cationic DOTAP lipid with its quaternary ammonium headgroup enhances interactions with anionic lipids of target mesenchymal stem cells through electrostatic association.^97^ Different incubation times (1, 4, and 24 h) and liposome concentrations (125, 250, 500, 1000 µM lipid) were trialled in order to investigate the time and dose dependency on viability, proliferation and differentiation. An optimised dose (125 µM) and time (4 h) were found to correspond to an intracellular Gd load of 30 ± 2.5 pg Gd cell^−1^, with no associated adverse side effects observed *in vivo*.

Lipids possessing negatively charged head groups include phosphatidylglycerol (PG), found in cell membranes and as a pulmonary surfactant, and cardiolipin (CL), found in the inner mitochondrial membrane.^98^ Other examples of anionic lipids include phosphatidylserine (PS), phosphatidylinositol (PI), and phosphatidic acid (PA). Anionic PS is a biological signalling component capable of activating positively charged signalling proteins *in vivo*. PS residues are also known to be present in apoptotic cells, for the purpose of macrophage recognition and subsequent phagocytosis.^99^ Maiseyeu *et al.* exploited this biological recognition pathway in paramagnetically-doped liposomes comprised of PC, PG, rhodamine-modified DOPE, cholesterol, sulfonamide functionalised Gd–DTPA and PS for the purpose of imaging atherosclerosis by mimicking apoptosis.^100^ PS presence enhanced macrophage uptake compared with analogous non-PS containing liposomes, with a concomitant, cellular internalisation driven, enhancement in image contrast (*T*_1_ = 772 ms to 467 ms; 1.5 T).

### 3.3. Lipid tail engineering

In addition to phospholipid head-groups, phospholipid tails can also be modified, influencing membrane properties controlled by the tail saturation and/or length, such as hydrophobic tail packing structure and inherent permeability/water diffusivity.^101^ Of course, this is paramount to consider within the context of responsive liposome-based MRI CAs, where overall relaxivity in core-doped structures is dominated by transmembrane flux or propensity for disintegration. In cellular membranes, it is favourable to maintain membrane fluidity, with typical cell-forming membrane lipids (including PC, PE and PS) introducing kinks into the phospholipid tails arising from the presence of a *cis*-double bond.^102,103^ POPC is known for its utility as a biomimetic phospholipid, possessing mixed saturation in its lipid tails, prohibiting tight packing and inflexible membranes due to the one saturated (straight) and one unsaturated (kinked) tail. In comparison, DOPC with two unsaturated tails enables fluidity and flexibility, whereas the conically shaped lyso-PC perturbs bilayer packing and enhances membrane permeability.^104^ The mixed acyl chains in soy-PE/PC are such that this is a popular choice as a drug delivery vehicle, being derived from a natural source.^105^

To understand how acyl chain length affects the permeability coefficient (a measure of water diffusivity across the lipid bilayer, measured in µm s^−1^) Lopez *et al.* performed a systematic study across 15 different lipids with varying acyl chain lengths (C14–C24), and between 1–4 double bonds at various positions (*e.g.*, *cis* Δ6, Δ9, Δ11 *etc*, where Δ refers to the position of the *cis* double bond in the alkyl chain) using droplet interface bilayers (DIBs).^106^ Unsurprisingly it was observed that as the number of double bonds increased from 1 to 4, the permeability coefficient increases from ∼100 µm s^−1^ to ∼300 µm s^−1^. Shorter acyl chain lengths were shown to exhibit increased water permeability coefficients due to the reduced thickness of the phospholipid bilayer.

In similar work which investigates the specific engineering of lipid tails to control relaxometric properties, Cressey *et al.* functionalised either a longer (C_18_) or a shorter (C_16_) phospholipid tail (termed LCA-1 and LCA-2) with Gd–DOTA to investigate how changing acyl chain length directly impacts bilayer phase transition temperature (*T*_m_, Fig. 4), and therefore cross-bilayer water exchange, the latter governing *r*_1_.^107^ The molar ratio of LCA-1 : LCA-2 was systematically varied to understand how changing the phospholipid proportions influenced packing density, *T*_m_, and cross bilayer diffusive water flux. A decreased acyl chain length produced a slightly more fluid lipid bilayer membrane, with a reduced phase transition temperature (*T*_m_). Dynamic light scattering (DLS) confirmed a hydrodynamic size of 100 nm for all formulations, with differential scanning calorimetry (DSC) traces confirming an increase in *T*_m_ from 34 °C to 41 °C as %LCA-1 increased from 0–100. *T*_1_ measurements at 9.4 T (and 298 K) displayed that as %LCA-1 is reduced from 75–0, *r*_1_ increases from 3.99 mM^−1^ s^−1^ to 5.37 mM^−1^ s^−1^.

**Fig. 4:**
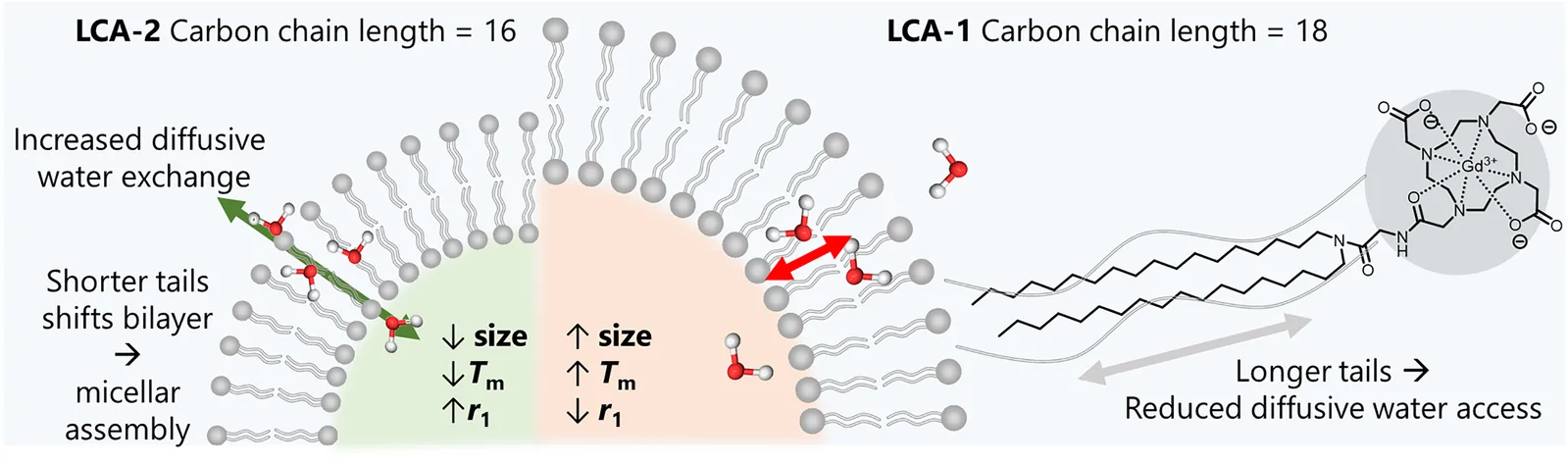
The length of the phospholipid tail is intrinsically linked to diffusive water access, with a thicker membrane reducing this.^107^ Liposomes comprised predominantly of lipids with longer acyl chains possessed a greater size, membrane transition temperature (*T*_m_), and reduced relaxivity. Naturally, the inverse is true, where liposomes with a majority composition of shorter chain lengths exhibit increased transmembrane exchange, attributed to the shorter phospholipid tails shifting the bilayer structure closed to a micellar assembly, increasing relaxivity.

To summarise, the characteristics of phospholipid heads and tails may be readily customised to modulate the downstream pharmacokinetic profile.^108^ This engineering directly impacts MRI contrast, as phospholipid tail modification can strongly influence bilayer packing density and cross-bilayer diffusive water flux. Phospholipid head group modifications also support the integration of peripheral MRI-active chelates.

## 4. Liposomal magnetic dopants

In order for liposomal constructs to promote protic relaxation, relevant chelates, coordination complexes, or nanoparticle-based dopants are, of course, required. These can be integrated in either a core-doped or periphery-doped manner (see Section 2.2). Below we set out the foundations required to introduce an MRI CA capability of liposomes prior to their application as effective environmentally-sensitive CAs, as detailed in Section 5.

### 4.1. Liposomes doped with paramagnetic coordination complexes

Gd- and Mn-based coordination complexes have been extensively studied for their internalisation within the intravesicular pool.^109–111^ Specifically, as GBCAs are used in ∼40% of all MRI scans (and ∼60% of neuro-MRI) to provide improved diagnostic clarity, a comprehensive, mechanistic understanding of Gd-based payloads within liposomal scaffolds is critical.^112^ Traditionally, due to the hydrophilic nature of [Gd(DOTA)]^−^ (defined by its highly negative octanol/water coefficient, log *D*_oct/PBS_ = −4.16), such hydrophilic chelates generally remain confined within the liposomal pool. Oliveira *et al.*, for example, have explored to what extent there exists any transient interactions between Dotarem® and POPC bilayers using molecular dynamics (MD) simulations to quantify the partition coefficients, permeation barrier, rotational correlation times and inner sphere residence time.^48^ The study established that, despite its highly hydrophilic nature, the chelate is able to interact, and even insert transiently, into the membrane, increasing its *τ*_R_ by over one order of magnitude compared with the analogous chelate in bulk solution, whilst maintaining an IS coordination number of 1. When inserted, the slow rotation of the membrane-associated GBCA elongates *τ*_R_ (bringing it closer to Larmor resonance frequency) resulting in a greater *r*_1_ value. Upon release into bulk solution, the re-established fast rotation diminishes *τ*_R_, with *r*_1_ expectedly decreasing. However, owing to the low calculated partition coefficient *K*_P_ = 1.5 (where *K*_P_ ≫ *D*_oct/PBS_), the overall effect on *r*_1_ is small, as the dominant form of [Gd(DOTA)]^−^ is the chelate form encapsulated in the liposomal pool. This study highlighted how observed relaxivity values can be understood through a quantification of the associated correlation times (*e.g.*, *τ*_R_).

Beyond GBCAs, there exists a range of *T*_1_ and/or *T*_2_ active metals (such as Fe-, Mn-, Dy- *etc.*) that have been integrated into liposomal scaffolds (Fig. 5), in the form of simple coordination complexes and chelates (including examples of *q* = 0 agents).^113,114^ Such alternatives have been investigated, because, despite the widespread use of native GBCAs in both clinical and research settings, they suffer from rapid renal clearance, low specificity, and toxicity.^115^ The latter is largely due to the risk of disease associated with gadolinium retention (*i.e.*, nephrogenic systemic fibrosis) which poses a greater risk to renally impaired patients.^116^ Additionally, GBCAs with unfavourable thermodynamic/kinetic stability profiles can de-chelate to release free Gd(iii), which can then bind to structures such as proteins, calcium channels, citrates or phosphates in the blood or extracellular environment, resulting in the observed toxicity.^117,118^ There is, then, significant translational value in designing kinetically inert scaffolds, alternative metal centres, and liposome integration to alleviate these issues.^119^

**Fig. 5:**
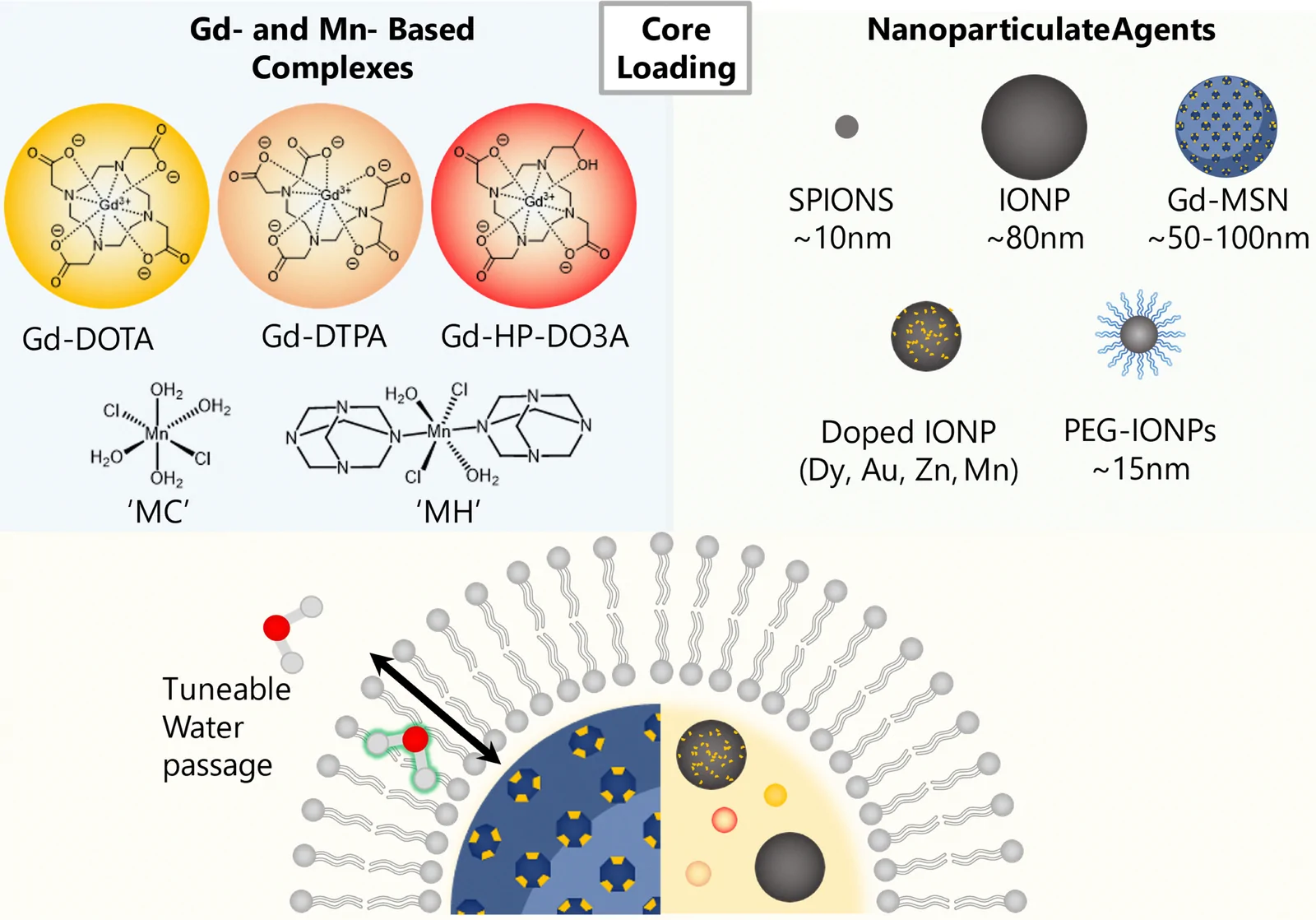
A representative range of chelates/nanoparticulate materials that can be encapsulated within the liposomal scaffold. These include clinically utilised Gd–chelates such as Dotarem® (Gd–DOTA), Gadoteridol (Gd–HP–DO3A), and Magnevist® (Gd–DTPA), in addition to Mn–chelate alternatives. Here, MC refers to MnCl_2_ and MH refers to Mn-hexamethylenetetramine. A range of (doped) iron oxide based structures of varying sizes, from SPIONs (10 nm) to IONPs (∼100 nm), including PEGylated IONPs (PEG–IONPs), can be internalised within such liposomal structures as well as more complex nanoparticulate structures, such as Gd-doped mesoporous silica nanoparticles (Gd–MSNs).

Early examples involved the oral liposomal formulation of MnCl_2_, employed for imaging of gastrointestinal issues (*e.g.* LumenHance®).^120,121^ However, such complexes suffered from poor stability *in vivo*, with *ca.* 40% of Mn(ii) released into the liver and spleen, attributed to the weakly coordinated metal within the complex possessing the incorrect coordination geometry. Despite this, Sguizzato *et al.* encapsulated MnCl_2_, denoted MC, and Mn–hexamethylenetetramine, MH, at a loading of 0.5 mM within liposomes comprised of soy PC, cholesterol and sodium lauryl lactylate (SLL) in a 4 : 2 : 1 molar ratio.^122^*r*_1_ values (1.5 T, 293 K) of 7.3 mM^−1^ s^−1^ (MC) and 7.7 mM^−1^ s^−1^ (MH) were reported respectively displaying good contrast due to the improved relaxivity *c.f.* Gd–DOTA (3.9 ± 0.2 mM^−1^ s^−1^ at 1.5 T, 275 K). This was attributed to association of the Mn(ii) centre not only with its coordination sphere, but also the inner/outer surface of the liposomal nanocarrier, elongating *τ*_R_.

Mn(ii)–chelates, as opposed to simple coordination complexes (such as MnCl_2_) represent more kinetically stable metal forms.^123^ In examples of this, Mangafodipir (Teslascan), comprised of Mn(ii) chelated by dipyridoxyl diphosphate (DPDP), has been employed for liver-specific imaging due to efficient uptake by hepatocytes.^124^ The encapsulation of these Mn(ii)–chelates within liposomal structures provides a further benefit in terms of limiting unnecessary accumulation in the heart and kidney compared with their non-encapsulated counterparts.^125^ Chen *et al.*, for example, have demonstrated that novel Mn(ii)–carbamazepine and valproate chelates, when encapsulated in 0.2 µm soy PE/cholesterol liposomes, effectively facilitate a dynamic assessment of the metabolic pathway and associated biodistribution by *T*_1_–weighted MRI.^123^ As this work was designed for the effective visualisation of epilepsy treatment, there was a specific emphasis on tracking the accumulation of Mn–chelate doped liposomes with a co-encapsulated antiepileptic drug payload in the brain tissue. In this study, the greatest *T*_1_–weighted signal was observed in the brain tissue 12 hours post tail vein injection, demonstrating by *T*_1_–MRI that this liposomal formulation can effectively cross the blood–brain barrier, promoting the repair of damaged brain tissue, without detectable toxicity.

The encapsulation of alternative Mn(ii)–chelates has been investigated for the purpose of characterising (and optimising) associated MRI contrast and *in vivo* biodistribution characteristics in a systematic study by Mulas *et al.*^126^ Here, three amphiphilic Mn(ii) complexes chelated by an EDTA derivative or two similar 1,4-DO2A structures were entrapped within a liposome scaffold, with the Mn(1,4-DO2A) chelate displaying high kinetic inertness and thermodynamic stability (*t*_1/2_ = 48.3 h at pH 7.4, compared with *t*_1/2_ = 0.076 h, pH = 7.4 for the parent Mn(EDTA)^2−^ complex), with an associated *r*_1_ of 2.1 mM^−1^ s^−1^ at 298 K and 0.47 T. Such moderate measured relaxivity is attributed to the two hydration forms *q* = 1 and *q* = 0 being present in an 87 : 13 equilibrium (*c.f. r*_1_ = 3.3 s^−1^ mM^−1^ at 298 K, 0.47 T for the *q* = 1 Mn(EDTA)^2−^ complex). To improve observed *r*_1_, Mn(HCDTA), Mn(DDO2A) and Mn(DH–DO2A) were synthesised, the latter two containing a C_12_H_25_ or a C_16_H_33_ alkyl chain respectively (phospholipid analogues) to achieve an elongated *τ*_R_. Subsequently, liposomes comprised of DPPC, DSPE–PEG2000 and Mn(ii)-complexes (20 mol%) exhibited *r*_1_ values (0.5 T, 298 K) of ∼17 mM^−1^ s^−1^ (Mn(HCDTA)), ∼16 mM^−1^ s^−1^ (Mn(DDO2A)) and ∼11 mM^−1^ s^−1^ (Mn(DH–DO2A)) respectively. To rationalise the observed elevated relaxivity values of the 20 mol% Mn(DH–DO2A) phospholipid-based nanoparticles compared with analogous Mn–chelates, a Lipari–Szabo adapted SBM relaxation model was employed. This enabled separate descriptions of both local and global rotation of the chelate-doped liposomes. In doing so, it was demonstrated that *r*_1_ was indeed augmented by the successful slowing of the local molecular rotation of membrane periphery-bound chelates.^127^

*T*_2_-weighted MRI contrast can be achieved in Fe-based architectures, such as haemoglobin (Hb), its deoxygenated form (deoxyHb, Fe^2+^), and methaemoglobin (MetHb, Fe^3+^) both of which have been explored as vascular contrast agents.^128,129^ Sakai *et al.*, for example, have encapsulated Hb within liposomes (termed HbVs) as an artificial red blood cell mimic.^130^ This was further expanded in a consideration of how MetHbVs could be explored as an intravascular CA of good biological compatibility, metabolic performance, and blood retention time (∼16.7 h).^131,132^ An observed 2.4× increase in *r*_1_ (and similar *r*_2_) for Met–HbVs was observed compared with the analogous haemoglobin-doped liposomes, with this assigned to high liposomal concentrations and rotation-restricting interactions with phospholipid heads. However, their comparatively low reported relaxivity values (*r*_1_ = 0.61 mM^−1^ s^−1^, *r*_2_ = 1.89 mM^−1^ s^−1^) demonstrates that further optimisation is required.

In a consideration of extending from simple paramagnetic chelate/complex examples, nanoparticulate structures offer much in improving intrinsic relaxometric capabilities, specifically through elongation of inherent rotation and diffusive correlation times.^65,133^ Consequently, they are commonly incorporated within the liposomal interior and/or bilayer membrane as detailed in Section 4.2.

### 4.2. Nanoparticulate dopants

There is a range of nanoparticulate configurations that support highly effective MR imaging, either as the native inorganic scaffold,^134^ or combined into hybrid organic–inorganic forms (*i.e.*, by PEGylation or liposomal encapsulation).^135^ Such entities include lanthanide-doped mesoporous silica nanoparticles (Ln–MSNs)^136^ and calcium phosphates (Ln–CaPs),^137^ iron oxide nanoparticles IONPs (see Section 4.3),^138^ and doped quantum dots (QDs).^139,140^ NP-based MRI CAs can offer significantly enhanced (∼5–10× and above) relaxivity compared to their analogous chelates.^141,142^

MSNs have, for example, been widely investigated for clinical applications in both drug delivery and medical imaging due to their highly porous nature. Encapsulation of MSNs within liposomal architectures has been demonstrated to improve cellular uptake when compared to native Gd–MSNs, and, when integrated with peripheral targeting ligands, enable specific localised treatment.^143^ Liposomal encapsulation of loaded MSNs also ensures payload protection from enzymatic degradation, avoids premature payload release, and masks any reactive groups on the MSN interface to further enhance their immunogenicity and associated biosafety.^144^ Gd integration into these MSNs generates *T*_1_ CAs that are ∼20x more efficient than traditional molecular variants by virtue of restricted chelate rotation afforded by the comparatively large and rigid scaffold, and the strong, diffusion-modulating effects of the polar silanols, originating from strong hydrogen bonding (*r*_1_ = ∼30 mM^−1^ s^−1^).^145,146^ In an example where decorated bilayer wrapped MSNs enable specific *in vivo* targeting (and imaging) Jin *et al.* developed a pro-apoptotic peptide entrapped within Gd-doped MSN pores, all encapsulated within a lipid bilayer comprised of DPPC and DSPE–PEG.^143^*T*_1_-weighted MR images were obtained, with the peripheral peptide supporting successful tumour cell entry and subsequent apoptosis.

Among all nanoparticulate dopants, liposome encapsulated IONPs, known as magnetoliposomes (MLs), are by far most extensively studied composite architectures. As such, we discuss them separately below.

### 4.3. Magnetoliposomes (MLs)

IONPs have been widely investigated within clinical imaging, therapeutic, and biosensing technologies, owing to their native and tuneable magnetic nature.^147^ For example, under an externally applied AC field, these properties support the generation of a locally hyperthermic environment, owing to the dissipation of energy as heat.^148^ Superparamagnetic IONPs (SPIONs) with an associated size of 10–100 nm primarily function as *T*_2_ CAs,^149^ whilst ultra-small SPIONs (size < 5 nm) have been employed as *T*_1_ agents, specifically as an alternative to GBCAs.^150–152^ As serum proteins are known to coat/foul the surface of native IONPs,^153^ there is considerable chemical/biochemical value in liposomal wrapping, rendering them non-fouling and improving both colloidal stability and *in vivo* circulation time. Furthermore, for unmodified IONPs, biocompatibility is a significant concern; ferumoxytol (clinically employed SPIONs) are known to potentially cause anaphylaxis, resulting in an FDA black box warning.^154^ An organic modification of the IONP periphery by either liposome encapsulation or PEGylation (to produce PUSIONPs, PEGylated Ultra-Small IONPs) shields the nanoparticulate payload from the immune system, minimising severe acute hypersensitivity reactions.^155,156^ PUSIONPs, for example, present improved vascular contrast and prolonged imaging windows (*r*_1_ = 6.20 mM^−1^ s^−1^ at 3.0 T) owing to enhanced blood circulation *c.f.* Magnevist® (*r*_1_ = 3.47 mM^−1^ s^−1^, 3.0 T).^157^ Liposomal encapsulation provides complementary advantages over PEGylation alone (*e.g.* PUSIONPS) by offering payload encapsulation/confinement which can be integrated alongside surface functionalisation strategies to impart both ‘stealth’ characteristics and the ability to tune/optimise observed contrast (see below).

Differences in local magnetic susceptibility as IONPs switch from a monodisperse to clustered state on liposomal encapsulation can be invoked to dramatically influence CA performance.^158^ Clustered IONPs exhibit greater *T*_2_ contrast, owing to their stronger net magnetic moments due to the collective magnetic behaviours of several particles within close proximity, compared to their monodisperse counterparts.^159^ Such magnetic clustering effects have been demonstrated by Zhou *et al.*, where encapsulation of PUSIONPs in liposomes exhibited a concentration-dependent effect as increasing local PUSIONP concentration could drive the associated relaxivity as high as 288.0 mM^−1^ s^−1^.^41^ This approach is typically employed in the design of responsive CAs, where *T*_2_ (and by extension *r*_2_/*r*_1_) can be modulated through release of encapsulated IONPs into solution on bilayer fragmentation (as discussed in Section 5.1).

Confining hydrophobic SPIONs within a bilayer membrane is therefore an effective method for promoting cooperative magnetisation effects, generating *T*_2_-weighted MRI CAs. In an example by Martínez-González *et al.*, either hydrophilic or hydrophobic SPIONs were loaded into liposomes of varying lipid composition in a systematic study to unveil which lipid composition and SPION incorporation strategy produced the greatest relaxivity value.^160^ Specifically, hydrophilic SPIONs (controlled by surface chemistry) were incorporated within the liposomal cavity, with hydrophobic SPIONs embedded/loaded within the liposomal membrane. The latter possessed improved relaxometric properties over the former, attributed here to an established high local (iron) concentration. The greatest relaxivity values were observed for hydrogenated 1,2-Dimyristoyl-*sn*-glycerol-3 phosphatidylcholine (h-DMPC, *r*_1_ = 9.1 mM^−1^ s^−1^, *r*_2_ = 1282 mM^−1^ s^−1^ at 7.0 T) and largest magnitude *r*_2_/*r*_1_ observed for oleoyl–DMPC–PS or oleoyl–DOPC–PS (∼1000 for both).

It is known that other paramagnetic centres can be used in combination with IONP-based CAs. For example, Govindharaj *et al.* reported Dy(iii)-doped IONPs encapsulated within liposomes comprised of soy PC and cholesterol (4 : 1).^161^ Interestingly, Dy-doping was demonstrated to enhance *r*_2_ from 1.77 to 8.97 mM^−1^ s^−1^ as a result of increased paramagnetic Dy(iii) doping. In comparison, Mn-doped IONPs (15 nm) of *r*_2_ = 746 mM^−1^ s^−1^ (*r*_2_/*r*_1_ = 16) have been encapsulated in biomimetic liposomes and synthetic hybrid vesicles respectively. Rocha *et al.* proposed that reducing the core diameter of the Mn–IONPs could allow CAs with dual *T*_1_–*T*_2_ properties to be formed, by specifically tuning packing/clustering in order to modulate their *r*_2_/*r*_1_ ratio (similar to work by Zhou *et al.*).^41,162^ Significantly improved *r*_1_, *r*_2_, and *r*_2_/*r*_1_ values were displayed across encapsulation of Mn–IONPs within both synthetic liposomal structures (and distinct biological cell membranes, such as erythrocyte/melanoma/glioblastoma fragments) when compared with ProHance (Gadoteridol, *r*_2_/*r*_1_ = 1.18). The greatest *r*_2_/*r*_1_ (37.9) was achieved for ML5.0_RBC (erythrocyte fragments, *r*_2_ = 445 mM^−1^ s^−1^). This was closely followed by *r*_2_/*r*_1_ = 35.9 for ML5.0_GL261 (glioblastoma fragments, *r*_2_ = 278 mM^−1^ s^−1^) showing significantly improved *T*_2_ contrast distinction compared with prior work. This work represents an interesting utilisation of biomimetic MLs formed from the coating of IONPs with cancer cell membranes, as opposed to synthetic lipid bilayers.

To conclude, the liposome scaffold can be carefully designed with a variety of core/bilayer dopants. Both core-doped and periphery-modified liposomal scaffolds are well suited in the generation of environmentally-responsive contrast agents. This is due to their distinct relaxometric ‘off’ and ‘on’ states, which report on the water accessibility to paramagnetic centres and/or molecular tumbling rates. Alternatively, contrast can be modulated through the tuneable proximity of IONPs (clustered/monodisperse) modulating *T*_1_*vs. T*_2_ dominance. The following Section elaborates on these concepts further.

## 5. Engineering responsive MRI CAs

When aligned with a specific microenvironment (*i.e.*, abnormal acidity/elevated temperature *etc.*), information relating to *in vivo* disease status can be acquired through an associated relaxometric switch. For example, cleavage of the covalent bond to the periphery-appended MRI active moiety, or bilayer lysis in the presence of triggering conditions (such as low pH, high temperature, oxidative stress or specific wavelengths), biases local chelate rotation (*τ*_R_ decreases), with a concomitant reduction in relaxivity. In comparison, an initial ‘off’ state can be retrospectively switched ‘on’ due to a change in the effective ‘proxy’ *τ*_M_ which reports on an increase in the size of the effective water pool available. This water pool can be effectively modulated by destructive, or non-destructive approaches (Fig. 6). Accordingly, contrast enhancements can be achieved in either an irreversible (Section 5.1), or reversible (Section 5.2) manner.

**Fig. 6:**
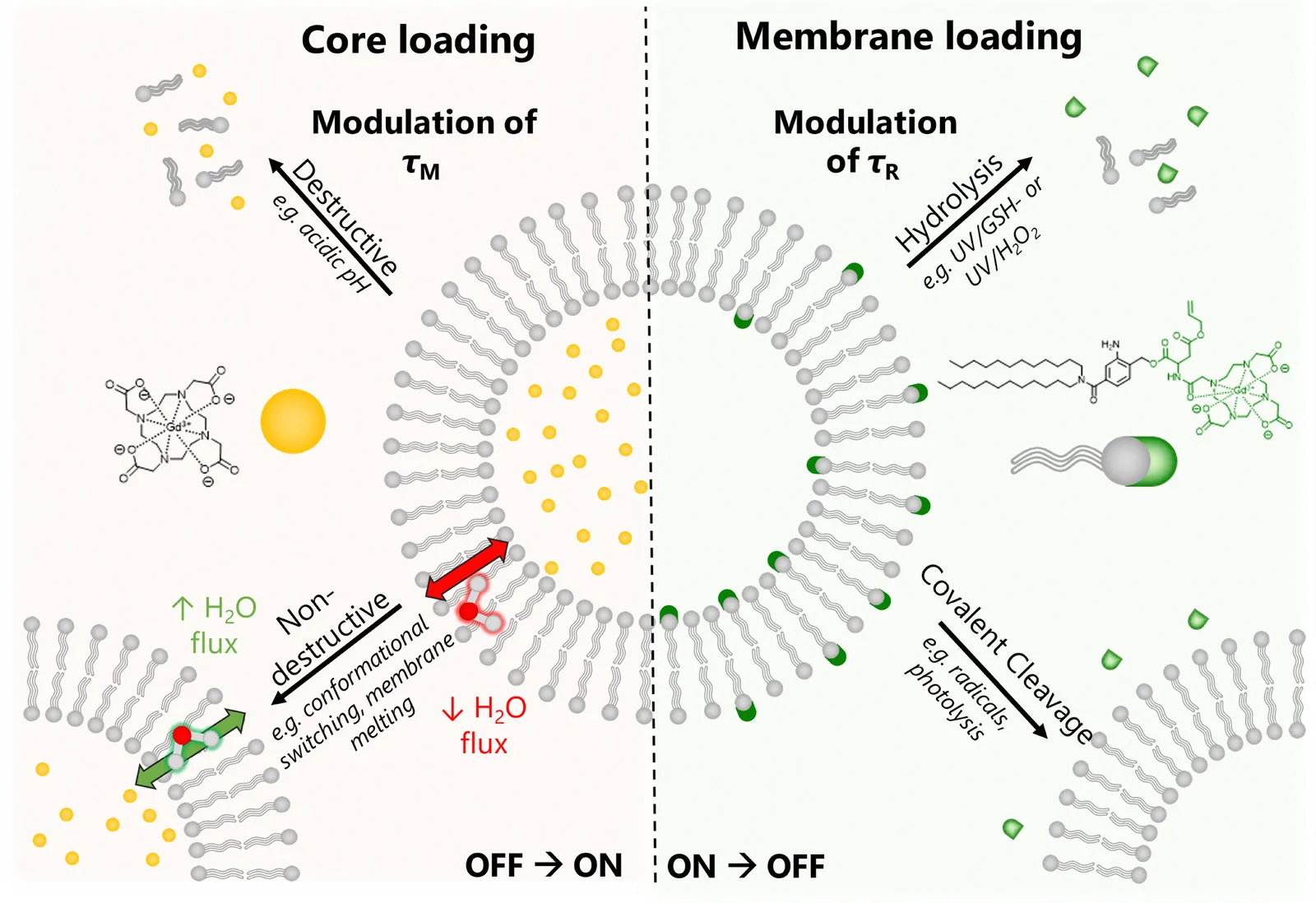
Depending on the location of the MRI active moiety, (appended to the structural periphery or internally encapsulated), either an MRI turn ‘off’ or turn ‘on’ can be achieved in response to external stimuli such as UV or heat. For example, modulation of a ‘proxy *τ*_M_’ from an initially MRI silenced state, achieved by restoration of specifically limited diffusive bilayer water flux, results in an MRI turn ‘on’ response. Typical stimuli which can modulate bilayer water exchange include bilayer ‘melting’, conformational switching of embedded motifs, or integration of ionophores. In contrast, degradation of the liposomal structure, in the case of appended chelates, results in an MRI turn ‘off’ response by a significant increase in the molecular tumbling rate, *τ*_R_. Modulations in *τ*_R_ generally result from covalent cleavage of GBCAs which are tethered to phospholipid head groups, such as in the presence of radicals, or photolysis (UV).

### 5.1. Irreversible liposome-mediated contrast modulation

Bilayer hydrolysis is generally associated with an MRI ‘turn on’, whereby an initially volume-capped diffusive water access to the paramagnetic material is re-established upon payload release, engineered to be in response to a specific stimulus.^163^ Membrane destruction has, for example, been achieved using methods that include ultrasound (sonosensitivity), typically applied for responsive drug delivery.^164,165^ US triggered disruption has, for example, been employed with Gd-doped liposomes by Quattrociocchi *et al.* to mimic liposome rupture *in vivo* with a > 4000% increase in *r*_1_.^166^ Here, 300 mM of Gadoteridol (Gd–HP–DO3A) was encapsulated within liposomes composed of 95 : 4 : 1 DPPC, DSPE–PEG2000 and maleimide functionalised DSPE–PEG2000 (by mol%). These liposomes were then decorated on the periphery with a targeting peptide specific to a protein commonly overexpressed in tumour cells (carbonic anhydrase IX, CAIX) by click chemistry. Prior to sonication cycles, the initial *r*_1_ (7 T) was measured at 0.1 mM^−1^ s^−1^, with the native relaxivity of Gadoteridol restored upon rupture (4.3 mM^−1^ s^−1^).

Triggered mechanical changes within paramagnetic liposomes can also be leveraged; here resonant contrast can be engineered to ‘switch’ on exposure to biologically relevant species (Ca^2+^,^167^ free thiols/radicals,^168^ trypsin,^169^ and folate^170^), enabling their detection.^32^ These effects are, specifically, facilitated by triggered changes in liposome aggregation state,^169^ or correlation times of bilayer tethered paramagnetic chelates. In an example of the latter, Garello *et al.* designed a Ca^2+^-sensitive agent by incorporating a modified amphiphilic ligand capable of binding Ca^2+^ within a DPPC and DSPE–PEG2000 based liposomal membrane (10 : 85 : 5 molar ratio).^167^ Integrating the Ca^2+^-responsive linker adjacent to the bilayer tethered Gd–chelate enabled rotation away of a single side arm carboxylate from the paramagnetic ion on Ca^2+^-binding, driving a hydration state change (*q* = 0 to *q* = 1) at the paramagnetic ion. Additionally, Ca^2+^-binding was proposed to cause rigidification of the linker, and a tighter mechanical coupling of the Gd centres with the slower rotating particle (increasing *τ*_R_). These two effects combined here to produce a substantial associated increase in *r*_1_ (7.3 mM^−1^ s^−1^ to 38.1 mM^−1^ s^−1^; 298 K, 0.5 T).

Liposomes have also been primed to undergo lysis when exposed to biological agents such as peptides or enzymes which are able to trigger cargo release (either paramagnetic and/or therapeutic).^171^ In an example of peptide-triggered release of Gd–DOTA from liposomes, Jian *et al.* pioneered a single point inversion recovery method (signal acquired at a singular fixed inversion time post 180° RF pulse) to report *T*_1_ relaxivity enhancements upon peptide insertion based on relaxation rate analyses (*R*_1_) rather than relaxivity (*r*_1_) values to avoid the need of determining [Gd(iii)].^172^ Incorporating a membrane-lytic antimicrobial peptide at a peptide-to-lipid ratio of 1 : 50 resulted in membrane lysis, increasing *R*_1_ from 0.287 s^−1^ to 0.698 s^−1^. In an example of enzyme triggering, Cheng *et al.* reported the release of encapsulated Gd–chelates from 100 nm liposomes comprised of 90 : 10 DPPC : DSPE–PEG2000 using phospholipase A_2_ enzymes (PLA_2_).^173^ After 1 hour of PLA2 incubation, a 45% decrease in *T*_1_ relaxation time was measured (higher *T*_1_-weighted signal), attributed to the enzymatic hydrolysis of the bilayer causing release of GBCAs into bulk solution upon cleavage of the phospholipid sn-2 ester bond.

In addition to enzymatic or peptide-mediated approaches, light-responsive liposomes have also been studied for remote-controlled/externally-regulated MRI contrast. Reeßing *et al.*, for example, have designed a light-responsive liposomal agent by tethering a Gd–chelate, linked by a photocleavable *ortho*-nitrobenzyl group to the phospholipid head within the liposomal architecture.^174^ Irradiating for 60 minutes at 400 nm resulted in a decrease in *r*_1_ of 49%, from 10.7 mM^−1^ s^−1^ (on) to 5.2 mM^−1^ s^−1^ (off) at 0.2 T. Despite this effective generation of responsive contrast, MRI turn ‘off’ approaches are clinically less valuable than turn ‘on’, as signal loss can be misinterpreted. For example, reduction in contrast could be as a result of artifacts *in vivo*, such as variations in tissue susceptibility or inhomogeneities in the applied magnetic field, rather than the desired specific stimuli-response. In an example of a light-responsive *T*_1_ contrast turn on, Fernández-Méndez *et al*. encapsulated ultra-small IONPs within liposomes comprised of a 9:1 molar ratio of DPPC and 1-stearoyl-2-hydroxy-*sn*-glycero-3-phosphocholine (Lyso-PC).^175^ At 1.5 T, the initial *r*_2_/*r*_1_ was evaluated at 2.4 for the ultrasmall IONPs (*r*_1_ = 7.0 ± 0.1 mM^−1^ s^−1^, *r*_2_ = 17.2 ± 0.3 mM^−1^ s^−1^, *T*_1_-weighted) which upon subsequent liposomal encapsulation gave *r*_2_/*r*_1_ = 15.6 (*T*_2_-weighted). Varying percentages of DSPE-PEG-2000 were incorporated after ML formation to improve colloidal stability and antifouling properties *in vivo*. Prior to laser exposure (NIR-II, 1064 nm), the elevated local concentration of encapsulated IONPs results in *T*_2_-dominant behaviour due to augmented local magnetic susceptibility effects. In order to simulate this rupture at the tumour site, a detergent (Triton X-100) was used to test how *r*_2_/*r*_1_ varies upon lysis. An *r*_2_/*r*_1_ = 2.7 was observed, consistent with a dynamic shift to the initial *T*_1_-weighted behaviour of well-dispersed IONPs upon degradation of the liposomal bilayer, with concomitant drug release of the encapsulated therapeutic, potentially contributing to tumour cell death.

Lipid compositions possessing an intrinsic sensitivity to acidic microenvironments would enable valuable prognostic information given that lowered pH is associated with a variety of diseases.^176^ DOPE, for example, is widely used as a ‘helper lipid’ (alongside pH-responsive cholesteryl hemisuccinate, CHEMS) in targeted gene/drug delivery applications, with a conical shape which forms an inverted hexagonal phase upon exposure to an acidic environment.^6,177^ For example, by tuning the ratio of DOPE : CHEMS : PEGylated DSPE, Shah *et al.* were able to enhance the anticancer efficacy of cisplatin through a mediated release of therapeutic solely in the acidic tumour microenvironment.^178^ Such formulations, whilst common in drug delivery of anticancer agents, are relatively scarce for pH-responsive MRI, and hence future theranostic agents (co-encapsulated paramagnetic material alongside therapeutic cargo) could utilise such DOPE : CHEMS based scaffolds.

Although destructive activation of contrast in paramagnetically doped liposomal scaffolds is a facile approach to generate significant relaxometric change, lysis releases diagnostic cargo which may then have off-target, adverse interactions *in vivo*. Furthermore, such methods possess inherent irreversibility in response due to the hydrolytic mechanistic nature.^179^ Hence, in aspiring to non-destructive stimuli-responsive contrast, native water permeability is typically modulated. In such cases the liposomal platforms generally exhibit more muted switches in relaxivity compared with hydrolytic approaches, as the bilayer (post switch) continues to impose a diffusive water barrier to the encapsulated paramagnetic cargo. Nevertheless, the significant benefit of reversible activation of contrast potentially precludes off-target activation which imparts increased microenvironmental specificity.

### 5.2. Non-destructive liposome-mediated contrast modulation

For non-destructive stimuli-responsive MRI contrast leveraging modulations in water flux across intact bilayers, strategies range from thermosensitive phospholipid scaffolds which impart temperature sensitivity, to the incorporation of photo-switchable motifs which tune phospholipid tail packing,^180^ and, more recently, the incorporation of mobile ion carriers or ion channels.^49,181^ It is known that thermo-responsive bilayers operate by the concept of ‘membrane melting’, where lipids such as DPPC exhibit a 2000-fold difference in water permeability between the gel and liquid bilayer phase resulting from the temperature-dependent packing of two saturated C_16_ chains.^182^ Below 41 °C, DPPC exists in a gel phase, with significantly diminished cross-bilayer water flux across the rigidified membrane (MRI ‘off’). Above *T*_m_, the membrane transitions into a fluid liquid phase with enhanced associated water exchange (MRI ‘on’).^183^ Its gel-liquid phase transition temperature (*T*_m_) aligns closely with hyperthermic conditions (40–43 °C).^184^ To this end, DPPC has been used as a drug delivery vehicle to release cargo near its *T*_m_ for the purpose of inhibition/destruction of tumour cells.^185^

Temperature-dependent relaxivity imparted by DPPC has been observed in a number of SPION-based platforms. In an example of this, Lorenzato *et al.* employed a molar ratio of 100 : 33 : 27 : 7 (DPPC : HSPC : Chol : DSPE–PEG) with encapsulated ultra-small SPIONs to investigate the temperature-dependent nature on relaxivity.^186^ Upon heating, *r*_1_ increased from 1.6 ± 0.3 mM^−1^ s^−1^ to 4.6 ± 0.7 mM^−1^ s^−1^, owing to the increased transmembrane permeability of the liquid bilayer phase compared with the gel phase, with a concurrent decrease in *r*_2_ from 221.3 ± 15.3 mM^−1^ s^−1^ to 139.1 ± 4.6 mM^−1^ s^−1^, with significant relaxometric differences persisting even after the cooling period (irreversible change post heating cycle). This can be described as a non-destructive irreversible ‘membrane melting’, whereby control over membrane permeability (*i.e.*, water flux) exerts a measurable change in relaxometric response (see Section 2.2). As an alternative, PG, specifically 1,2-dipalmitoyl-*sn*-glycero-3-phosphodiglycerol (DPPG_2_), has also been used in thermosensitive liposome formulations, with a study by Hossann *et al.* investigating the encapsulation of 6 different clinically approved GBCAs with formulations comprised of 50 : 20 : 30 DPPC : DSPC : DPPG_2_.^187,188^ These liposomes were designed for MRI assisted interventional thermotherapy, exhibiting high stability in serum and ultrafast drug release and concurrent MRI switch “on” (Δ*r*_1_ = 3.7–7.2 mM^−1^ s^−1^ for non-ionic CAs) due to chelate release upon crossing the phase transition temperature *T*_m_.

Trans-bilayer water permeability can also be controlled and modulated not only through the choice of phospholipid which are susceptible to environmental response, but also by the introduction of further functional motifs such as photo-switchable molecules (*i.e.*, azobenzenes) which can be incorporated into phospholipid tails.^189^ Photo-isomerisation between the E- and Z-states changes the packing density of the phospholipid tails, reversibly altering the rate at which bulk water is able to cross the liposomal membrane. For example, Simon *et al.* developed a UV-responsive MRI probe, achieved by utilising Gadoteridol-doped liposomes, within which a photo-switchable azobenzene motif was incorporated into the fatty acid chain of the phospholipids (Fig. 7).^180^ In this particular work, trans-membrane water flux was directly quantified and demonstrated to vary between 0–0.01 cm s^−1^ (inversely proportional to membrane thickness),^190^ yielding *r*_1_ values that ranged from 0–3.8 mM^−1^ s^−1^. In the *cis* conformation (370 nm irradiation) the membrane permeability (and associated *R*_1_) is enhanced over two-fold, due to the enhanced bilayer water diffusivity compared with the *trans* conformation (associated wavelength = 460 nm), allowing MR contrast to be selectively switched ‘on’ and ‘off’ repeatedly over 3 cycles.

**Fig. 7:**
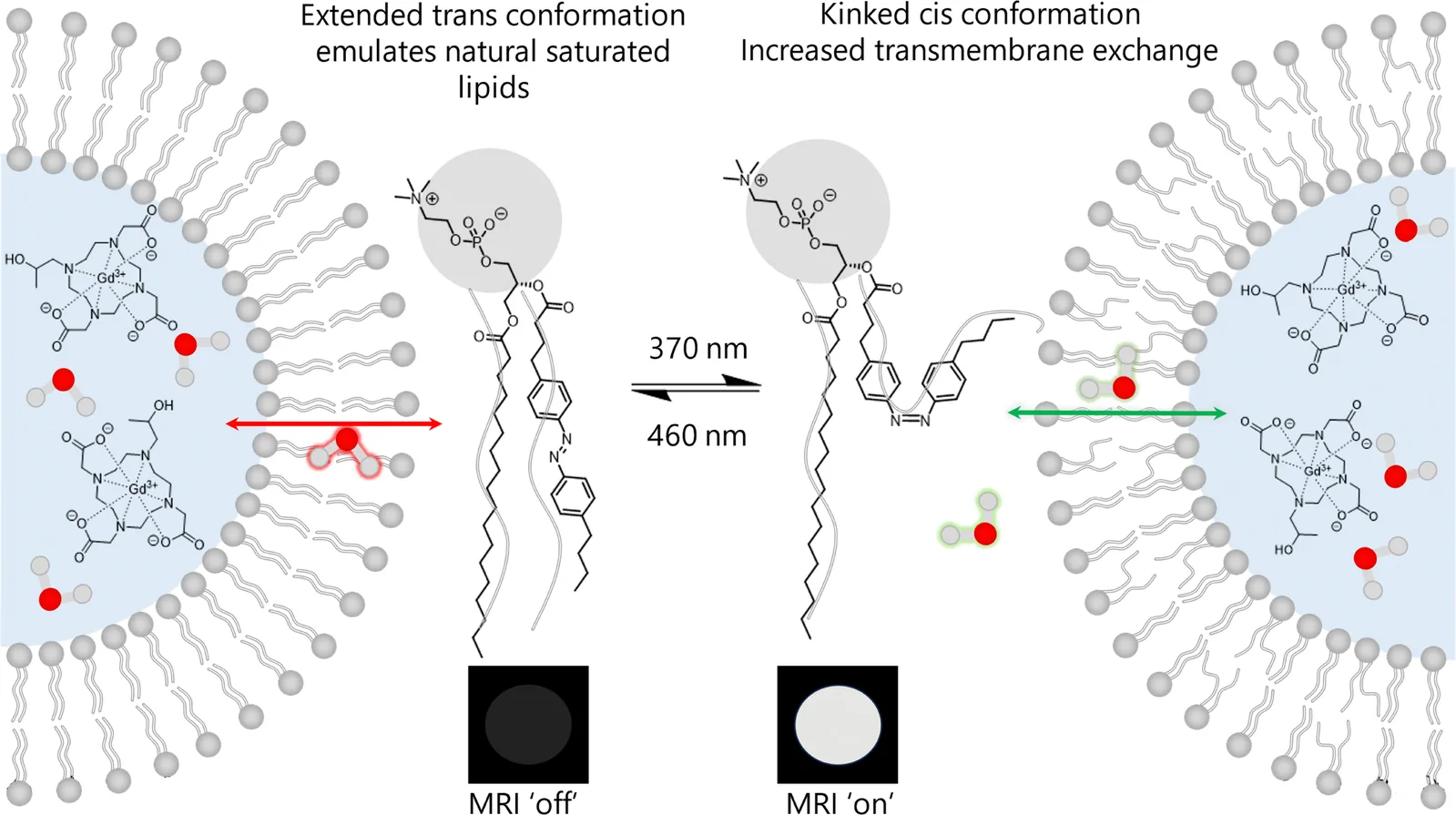
Strategies used for non-destructive/non-hydrolytic approaches to modulating MRI contrast can involve the utilisation of azobenzene motifs within phospholipid tails, which upon reversible *E*/*Z* isomerism, impart changes in cross-bilayer water exchange.^180^ This scaffold can enable stimuli-responsive contrast without releasing the encapsulated payload within the intraliposomal pool.

Rather than altering the chemical nature or structure of the membrane-forming phospholipids, engineered water channels such as gramicidin A, a pore-forming peptide, can be readily inserted into liposomes to enhance diffusive water access. To this end, Das *et al.* have demonstrated that the integration of gramicidin A enhances *R*_1_ across all vesicular membrane formulations as cross-bilayer water flux increases.^181^ Developing from this, they conceived a mechanism whereby modifying gramicidin A with desthiobiotin (dBt) at its exposed terminus would sterically block the entrance to the pore through association with streptavidin (Fig. 8). dBt binds streptavidin relatively weakly (*K*_d_ = 10^−11^ M) compared with biotin (*K*_d_ = 10^−14^ M), such that external addition of the latter competitively displaces streptavidin restoring water permeability. Biotin-sensitive responsive liposomal probes comprised of 100 nm membranes of varying DPPC : DPPG : POPC ratios were generated. Upon exposure to target (80 µM biotin), improved *T*_1_ weighted MRI performance was demonstrated by increases in *R*_1_ from 1.20 ± 0.03 s^−1^ to 1.34 ± 0.04 s^−1^. This approach was also applied to the sensitive detection of choline, an essential nutrient, acetylcholine precursor, and with a key role in lipid metabolism *in vivo*.^191^ Choline was found to block an anionic gramicidin A analogue (gA-COOH), decreasing water permeability and diminishing *R*_1_, with an associated *r*_1pore_ (mM^−1^ s^−1^) reported as ∼100, ∼70 and ∼30 for 0 mM, 10 mM and 100 mM choline respectively. Analogous liposomal nanoprobes with neutral, rather than anionic (containing a C-terminal carboxylate) gA displayed no significant modulation in observed MRI contrast.

**Fig. 8:**
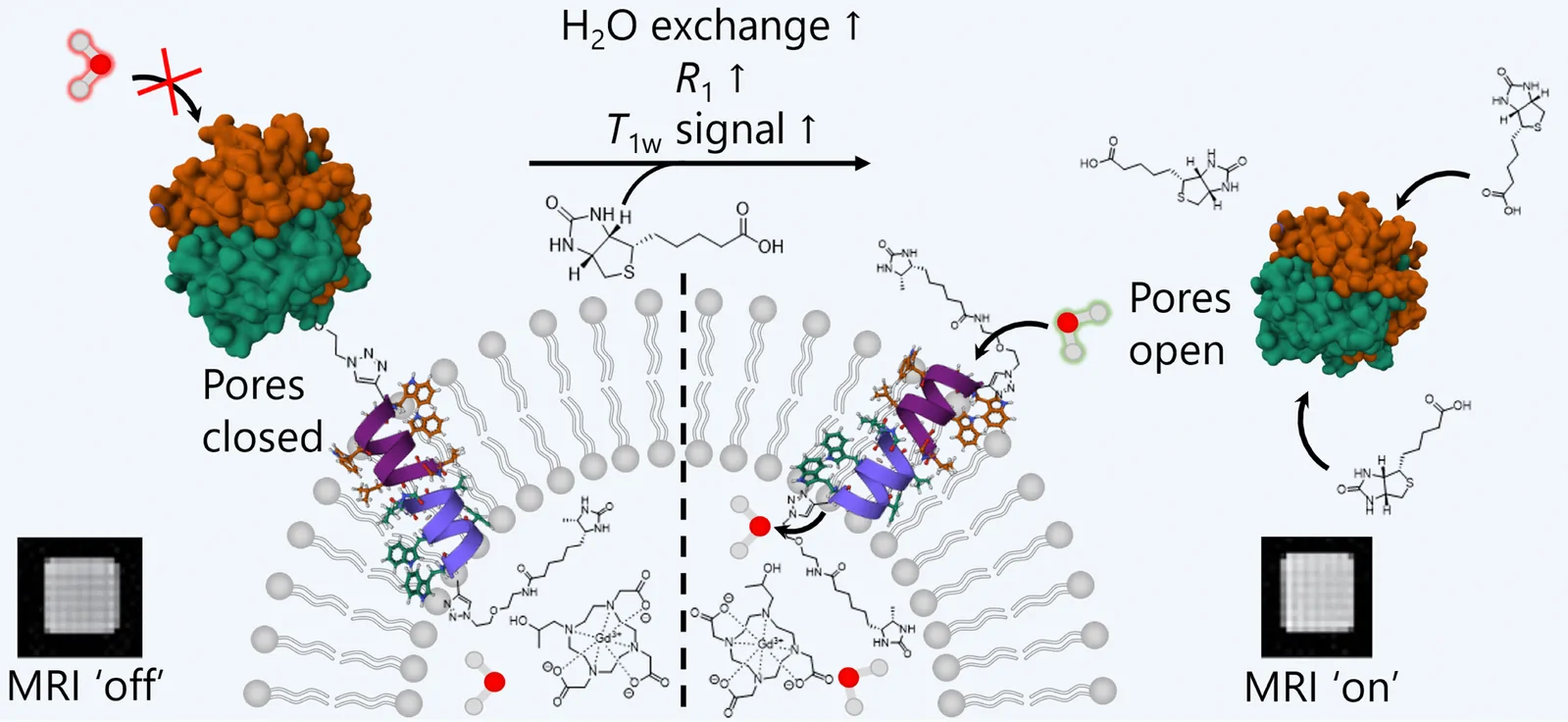
GramA is covalently bound to a desthiobiotin linker (gramA–dBt), rendering it susceptible to the presence of biotin. The presence of streptavidin (SA) blocks diffusive water access, such that water exchange is initially diminished, as too is the *R*_1_ and *T*_1w_ signal.^181^ SA is displaced in the presence of biotin, opening the pores and restoring diffusive water exchange.

Extending from the introduction of water channels into bilayer membranes, it has been demonstrated that some associated water flux can be controlled by the integration of mobile ion carriers or channels into lipid membranes which provide an energetically favourable pathway to the passage of ions.^192,193^ Increases in transmembrane water flux, afforded by the transfer of hydrated ions across the bilayer, to encapsulated Gd–chelates or Gd–MSNs can be leveraged to switch MRI contrast.^49^ In a mechanistically novel way of achieving ion-mediated switchable MRI contrast, Duncan and Ellis *et al.* utilised cholesterol modified POPC liposomes internally doped with Gd–MSNs to generate significant enhancements in *r*_1_ relaxivity (191%) upon the dual integration of K^+^ and Cl^−^ selective ion carrier species, specifically valinomycin and a tripodal thiourea-based structure (Fig. 9).^49^ Upon simultaneous transport of both ions (ionic symport), the carrier-mediated passage of both M^+^ and X^−^ across an initially highly hydrophobic bilayer is enabled. As these are hydrated, and maintain partial hydration during transport,^194,195^ water flux is greatly enhanced, supporting substantial switches in *T*_1_-weighted image contrasts. Associated variations in *r*_1_ were found to correlate with the selectivity of the cationophore and anionophore present.

**Fig. 9:**
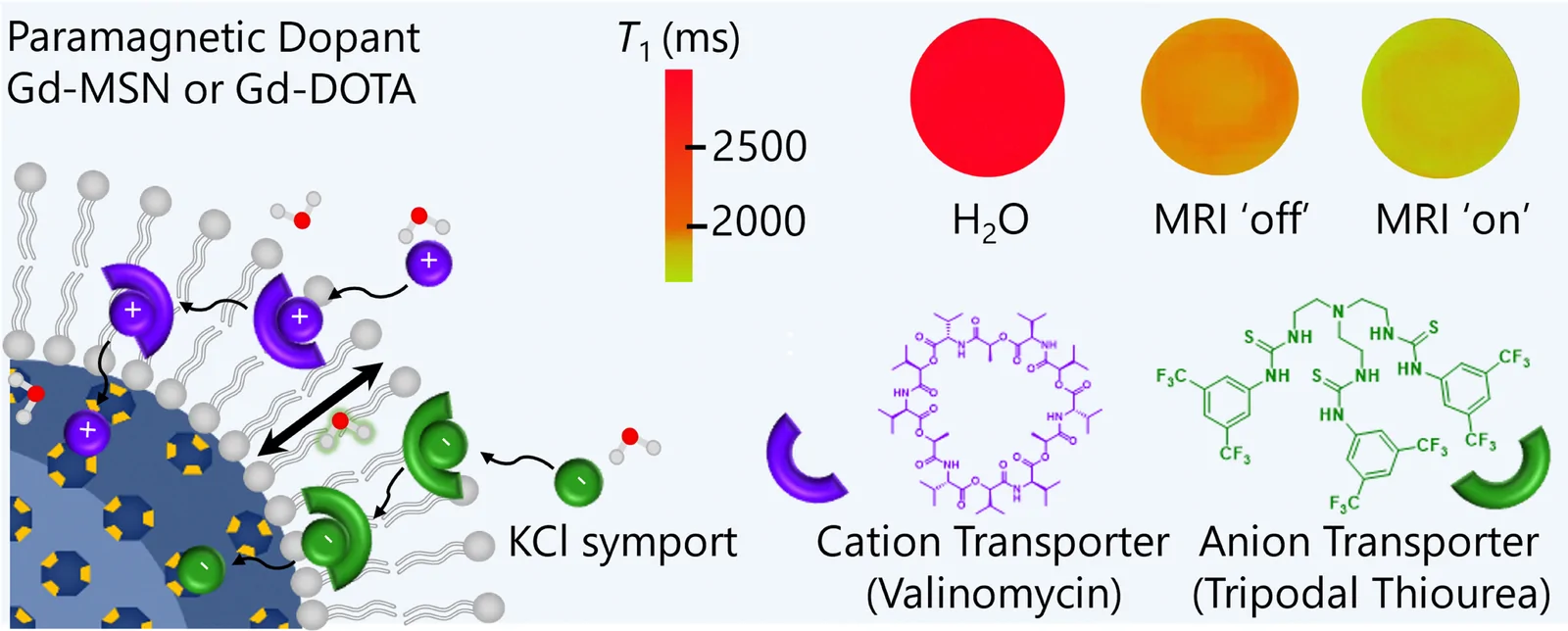
The integration of a chloride-selective tripodal thiourea anionophore, alongside the cationophore valinomycin, enables selective M^+^/X^−^ symport, with a significant enhancement in *r*_1_ for Gd–DOTA or Gd-MSN doped cholesterol-modified liposomes in specific ion presence.

Integration of an additional polymeric barrier between the bilayer and pore-confined Gd(iii) can provide a means by which ionophore promoted diffusive water flux can be even more dramatically switched. When these polymers are pH-sensitive, *i.e.*, p*K*_a_ crossing results in a pH-triggered conformational transition, significant relaxometric switching is known to occur (*e.g.*, Δ*r*_1_ ∼ 50 mM^−1^ s^−1^ at 1.5 T and Δ*r*_1_ ∼ 22 mM^−1^ s^−1^ at 3 T for pMAA–Gd–MSNs reported by Yuan *et al.*).^196^ Smith and Duncan *et al.* considered how this might be coupled to ionic flux across a liposomal membrane, as bilayers provide high energetic barrier to the passive passage of such charged species (and by extension water). In so doing, the group paired a pH sensitive polymer coat (pDMAEMA, p*K*_a_ = 6.4) to a transporter-activated bilayer; this directly coupled anion recognition and transport through the bilayer to an internal pH switch and large amplitude change in water flux to (polymer and bilayer) internalised Gd–MSNs.^197^ The approach of coupling ionophore-mediated specific solvated ionic flux with a pH-responsive polymeric water-gate was shown to be an effective means by which substantial *r*_1_ switches of ∼500% are generated (Fig. 10); this was confirmed to be the result of traversal of the p*K*_a_ of the encapsulated polymer upon H^+^/Cl^−^ symport, such that the internal polymer coat transitioned from a tightly wrapped (highly limited diffusive water access, MRI ‘off’) to more loosely associated form on the Gd–MSN interface (water flux enabled, MRI ‘on’).

**Fig. 10:**
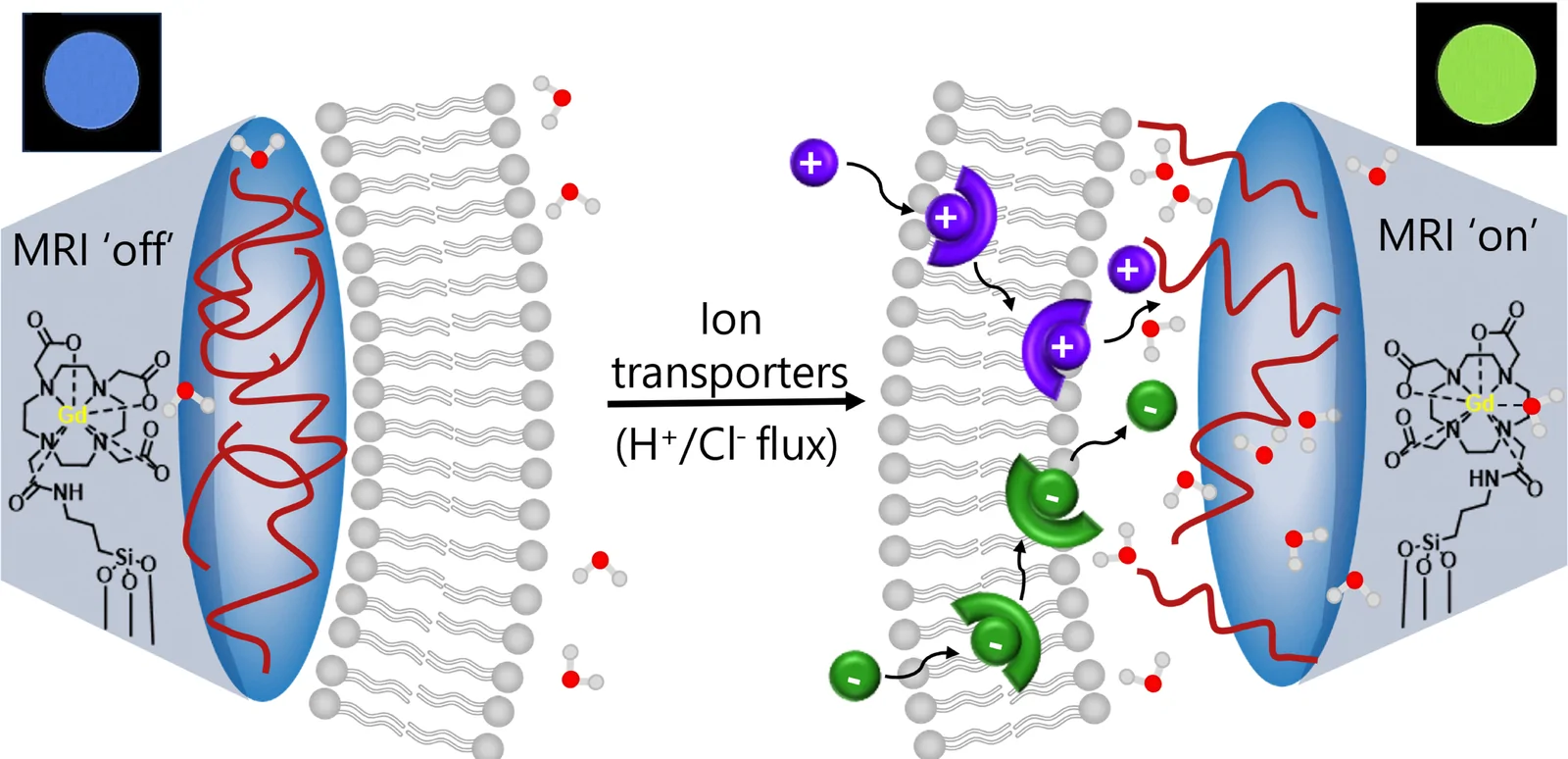
Coupling a pH-responsive polymer (pDMAEMA), possessing a p*K*_a_ that falls within a physicochemically relevant range (6.4), to specific ion transport events enables direct reporting on acidic extracellular pH. There is a substantial MRI switch ‘on’ (>500%) when H^+^/Cl^−^ is transported by a protonophore and a tripodal thiourea respectively. This is due to the swelling of the encapsulated polymer as the protonated tertiary amine groups electrostatically repel upon acidification, enhancing diffusive water access to the covalently tethered Gd–chelates within the MSN pore sites.

To summarise, stimuli-responsive MRI CAs enable a selective contrast enhancement in response to a specific environmental stimulus, and, as such, present themselves as valuable clinical tools supporting the imaging of pathological microenvironments.

Despite the benefits of responsive imaging, however, the fundamental issue of heterogeneous distribution *in vivo* is such that the contrast enhancement may be due to local concentration effects, rather than a specific MRI turn ‘on’ in response to a specific environmental stimulus, as relaxivity inherently depends on [CA]. Ratiometric probes have been developed to facilitate quantitative imaging, enabling observed contrast to be independent of agent concentration effects. This enables a more accurate evaluation of how these agents respond in a stimuli-responsive way to their immediate physicochemical environment, the principles and applications of which are discussed in the following Section.

## 6. Ratiometric MRI CAs and MRET

Incorporation of ratiometric architectures is, then, highly beneficial in generating stimuli-responsive MRI CAs,^198^ as localised areas of greater contrast distinction can be attributed to a specific environmental response, rather than passive accumulation effects. Typically, this is achieved through quantification of signal intensity ratios, such as *T*_1_/*T*_2_, or *r*_1_/*r*_2_ upon stimulus exposure. Examples include the ability to track MRI signal changes upon exposure to deviations in pH,^199^ reactive oxygen species (ROS),^200^ glutathione (GSH),^201^ hypoxia,^202^ enzymatic presence,^203^ adenosine triphosphate (ATP),^204^ ultrasound exposure, or light.^205^

In configurations where a constant baseline signal can be used as a reference for a responsive signal, concentration-independent analyses can be carried out. In one such liposomal example of this, Fowler *et al.* integrated 125 mM Dy–chelates (Dy–diethylenetriaminepentaacetic acid bismethylamide, Dy–DTPA–BMA) and 125 mM Gd–DTPA–BMA within LUVs comprised of 62 : 8 : 30 DSPE : DSPE–PEG2000 : cholesterol.^206^ In doing so, the Gd-based *T*_1_ signal was quenched through an exchange-limited internal water pool, whilst Dy–chelate promoted 
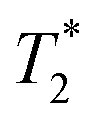
 (quantifying both innate transverse relaxation and additional magnetic field heterogeneity dephasing) facilitated monitoring of tumour accumulation through 
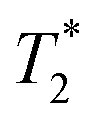
 relaxivity analyses (*r*_2_ = 0.079 s^−1^ mM^−1^). Peak accumulation within the tumour was observed at 48 h, highlighting both innate favourable circulation lifetimes and *in vivo* stabilities promoted by the liposomal architecture. Upon ultrasound mediated liposome rupture, *r*_1_ dramatically increased (5.04 mM^−1^ s^−1^), as water-exchange was re-established through release of the internal *T*_1_-active payload. By calculating the change in 
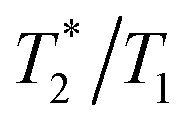
 (*i.e.*, Δ*R*_2_/Δ*R*_1_) between tumour accumulated and ultrasound-mediated release states, a concentration-independent quantification is possible (though not demonstrated here).

Aside from dual-probe ^1^H *T*_1_/*T*_2_ examples (as above), ratiometric approaches can alternatively integrate ^19^F, and/or paraCEST/diaCEST (chemical exchange saturation transfer) agents in generating internally referenced measurements.^207^ CEST agents function by indirectly imaging exchangeable solute protons at a different characteristic frequency to that occupied by bulk water protons.^208^ In liposomes, the internally encapsulated exogenous shift reagent is able to disturb the resonance frequency of the intraliposomal water protons by 1–5 ppm compared with the extra-liposomal protons. Consequently, a ratiometric analysis is enabled through chemical shift differences of the two unique water pools as characterised by diamagnetic, bulk magnetic susceptibility (BMS), and hyperfine contributions.^209–211^ The first reports of liposomal paracest CAs (LipoCEST agents) were by Aime *et al.*, whereby complexes such as Tm(iii)–DOTMA were encapsulated in 55 : 5 : 40 POPC : DPPG : cholesterol.^212,213^

In an extension of lipoCEST approaches to a stimulus-responsive configuration, Maruyama *et al.* produced thermosensitive liposomes comprised of 90 : 10 DPPC : polyoxyethylene (20) stearyl ether doped with Dy-, Tb- or Tm–DOTA chelates (20–80 mM) within the liposomal interior.^208^ Differences in chelate-stimulated intra- and extra-liposomal water signals enabled insight into the responsive nature of the contrast agent on exposure to different osmotic pressures. Specifically, increasing osmotic stress shrank the LUV architecture, leading to a difference in the BMS contribution, and therefore the reported chemical shift of the liposome agent, the effect most pronounced for the Tm-doped chelate. Whilst not inherently ratiometric, the co-integration of an additional baseline-generating chelate (*e.g.*, a *T*_2_-active motif), could support this.

Stimuli-responsive protic MRI signals can also be measured relative to a ^19^F ‘handle’ signal, offering a normalised quantitative readout. This has now been demonstrated in multiple reports based on polymer, inorganic, ionic liquid, and small molecule architectures.^214–219^ An added benefit is the high signal : noise of ^19^F reference signals due to the inherent lack of background ^19^F signal *in vivo.* Within the context of liposomes, the incorporation of ^19^F motifs is typically achieved by the integration of F^−^ and PF_6_^−^ agents within the central vesicular space. In one such example, Costanzo *et al.* formulated ultrasound-responsive liposomes comprised of DPPC with varying loading of F^−^ and PF_6_^−^.^220^ The greatest effects on observed ^19^F relaxation were measured for the smallest liposome size of 140 nm, indicating that a more confined internal water pool biases association between cationic lipids and anionic fluoride, increasing its tumbling time. Additionally, the largest decreases were observed for the liposomes doped with PF_6_^−^, with 89.4% and 99.6% reductions in ^19^F–*T*_1_ and *T*_2_ (150 mM loading) times respectively. Upon ultrasound triggered release, both ^19^F *T*_1_ and *T*_2_ relaxation times increase, providing a platform from which triggered drug release can be dynamically assessed. This work displays the natural potential that liposomes have to form future rationally engineered ratiometric contrast agents when ^19^F ‘handles’ are measured alongside a second, independent imaging signal.

In work by Langereis *et al.*, both ^19^F ‘handle’ probes and CEST agents were incorporated within temperature sensitive liposomes.^221^ The thermo-responsive liposomes were comprised of a 10 : 90 : 4 molar ratio of MPPC/DPPC/DPPE–PEG2000 doped with 50 mM NH_4_PF_6_ and Tm–HPDO3A respectively. At a temperature below *T*_m_, both dopants remain confined within the intravesicular pool, with a high protic CEST effect and a small ^19^F NMR signal, the ratio of which supports a concentration-independent readout. Upon heating to *T* > *T*_m_, lipid melting allowed for payload release into bulk solution, dramatically decreasing the protic CEST signal relative to ^19^F. Such studies highlight the relatively untapped potential of ratiometric liposomal MRI probes, in which simultaneous signal readout can provide additional information beyond traditional imaging, namely a quantification reflective of the local environment.

In comparison to the use of lipoCEST agents or baseline ^19^F ‘handles’, the MRET (magnetic resonance tuning) effect is a method by which MRI-derived *T*_1_ and *T*_2_ times can be tuned in a distance dependent manner to facilitate responsive imaging of physiologically relevant targets *in vivo*. *T*_1_ relaxivity is specifically tuned by modulating the distance between a superparamagnetic quencher (*T*_2_ active) and a paramagnetic enhancer (*T*_1_ active). When in close proximity, electron spin fluctuations of the enhancer are sufficiently slowed such that water proton relaxation is limited (*T*_1_ ‘off’). Upon separation, this effect is alleviated so that spin fluctuations can accelerate water proton relaxation (*T*_1_ ‘on’).^222^ In the case of ultrasmall SPIONs (1–5 nm), *T*_1_ and *T*_2_ relaxation effects can be achieved by a single agent; liposomally-confined SPIONs possess a clustering-driven quenched *T*_1_ activity such that *T*_2_ characteristics dominate.^223,224^ Upon destructive lysis, *T*_1_ activity is enhanced as individual SPIONs distribute in solution. However, MRET effects can be engineered to be ratiometric if one of the contrast-generating motifs acts as an internal reference signal; this is typically the *T*_2_-active agent. Liposomal scaffolds are primed for this, as they allow the facile confinement of a paramagnetic enhancer, such as Gd–DOTA, in close proximity to a superparamagnetic quencher (*T*_2_ active, Fig. 11).

**Fig. 11:**
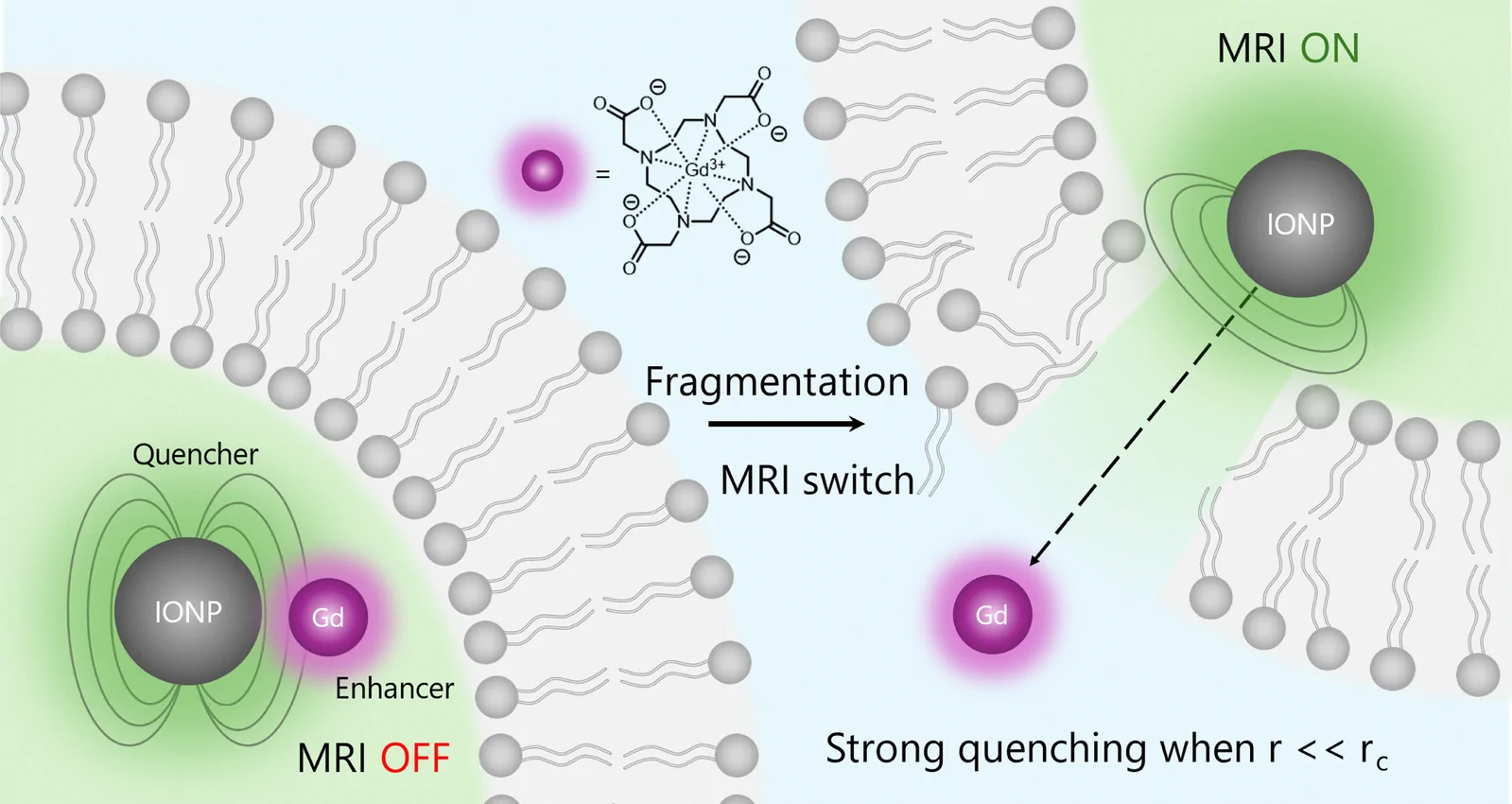
When the encapsulated enhancer (increasing *T*_1_ MRI signal) and quencher (suppressed *T*_1_ effect) are in close proximity, *T*_1_ MRI contrast is ‘OFF’. MRI contrast is switched ‘ON’ upon bilayer fragmentation due to increased spatial separation (greater than the critical separation distance, *r*_c_), generally achieved by a destructive approach, with a specific associated MRI turn ‘ON’ effect.

There are several examples of MRI probes which rely on the MRET mechanism for a ratiometric response, for example in the quantitative detection of hypoxia or GSH levels in tumour sites.^207^ In one, You *et al.* employed zinc-doped IONPs (Zn_0.4_Fe_2.6_O_4_@DMSA, quencher) and Gd–DOTA (enhancer) encapsulated within DSPE–PEG2000 doped, cholesterol modified POPC liposomes (2 : 4 : 20) to achieve an MRI ‘off’ state (*r*_1_ = 0.15 mM^−1^ s^−1^).^225^ Upon tumour cell entry, disruption of the liposomal bilayer through membrane fusion resulted in the establishment of a MRI ‘on’ state (*r*_1_ = 4.25 mM^−1^ s^−1^) as Gd–DOTA is released, and diffuses away from the quenching agent. Interestingly, this platform displayed ‘burst enhancement’ whereby the signal-to-noise ratio increases substantially after 2 hours, enabling clearer diagnostic distinction of the diseased tissue compared with healthy cells. This was attributed to selective enrichment within the tumour site and increasing separation between the enhancer and quencher over time.

MRET approaches, are advantageous in that they display high sensitivity and stability, circumventing the ‘always on’ state of non-stimuli responsive MRI probes.^207^ In the case of dual-modal MRET probes, the ability to continuously track *r*_2_/*r*_1_ presents as a powerful clinical tool for the detection of tumour progression, especially if the probe is designed to be ratiometric. Consequently, such platforms have already been utilised for detection and quantitative analysis of an upregulated biomarker MicroRNA-21 in breast carcinoma cells, due to its role in tumour development and progression.^226^ These agents, therefore, have been hallmarked as a next generation approach in bioengineering for improved clinical imaging efficiency.

## 7. Conclusions

Liposomal platforms enable the effective modulation of MRI characteristics through the facile tuning of size, available water pool, paramagnetic/superparamagnetic loading density, as well as the inherent clustering/elevated local concentration that results from cargo encapsulation. This represents a natural extension from the known applications of liposomes, where numerous formulations have already been approved clinically, owing to their advantageous biocompatibility and circulation times. Most importantly, the utilisation of liposomal scaffolds provides a defined water cavity/pool, which can be doped with a plethora of hydrophilic cargo. The incorporation of paramagnetic agents supports MRI activity, which, when introduced internally alongside other payloads, can concurrently facilitate a therapeutic (*e.g.*, anticancer) capability or further diagnostic (*e.g.*, PET) modality. In this review, rationally engineered liposomal platforms with incorporated MRI active motifs, have been highlighted; these enable specific responsive reporting on pH, temperature, enzymatic or local light fluctuations. Such reporting is mediated through differences in inherent relaxometric properties in response to a disease-specific or disease-relevant microenvironments.

In the case of internally doped architectures (which generally exhibit suppressed relaxivities), the underpinning feature behind MRI turn ‘on’ effects is the enhancement of water access to the paramagnetic entities. Such MRI turn ‘on’ responses are generally the preferred design of engineered responsive contrast, rather than ‘turn off’ approaches, owing to the lack of confusion with artefacts/blood clots of darker scan regions in clinical contexts. This alteration of water flux can take place by destructive/hydrolytic approaches, or non-destructively, making such classes of generating stimuli responsive liposomal MRI CAs either irreversible or reversible in nature. Already, a broad range of liposomal scaffolds have been tailored to respond to a variety of specific physicochemical environments which are hallmarks of disease states *in vivo*, such as low pH, elevated temperature and ROS presence, and has included a consideration of how contrast could be activated externally in patients, *i.e.*, by application of ultrasound or specific light presence which initiates a spatially confined MRI response. With this particular approach in mind, it is important to consider the practical nature of the wavelength used. Longer wavelengths such as NIR able to penetrate significantly deeper compared with shorter wavelength (UV) alternatives, and are more biocompatible. As such, engineering light-sensitive systems that are able to be triggered at longer wavelengths (NIR-I and NIR-II) is of particular importance.^227^ Customising organic photo-activatable moieties enables specific response upon exposure to different wavelength regimes, depending on the functional group employed. For example, boron-dipyrromethene (BODIPY) functional groups are desirable in photo-responsive applications, as they absorb in the red-NIR (near-infrared) region. Therefore, future work in photo-responsive liposomal MRI CAs should seek to employ more biocompatible wavelengths, despite the synthetic challenges associated with producing BODIPY-functionalised moieties.^228^

When considering possible avenues of future work, greater MRI switches are generally observed at lower field strengths (as defined by SBM theory).^229^ However, ultra-high-field (>7.0 T) imaging presents substantial benefits in terms of its improved spatial resolution and signal-to-noise ratio, enabling notable advances in the area of neuroimaging and functional mapping of neuronal activity.^230^ To this end, next-generation MRI scanners designed for such purposes are being built to achieve ultra-high resolution, increasing SNR and improving spatial encoding. Although suitable liposomal *T*_2_ CAs based on the incorporation of hydrophobic/hydrophilic SPIONs for MRI have been reported at 7 T, besides magnetoliposomes, ultra-high field examples of liposomal contrast are, thus far, limited.^160^ Future work should consider that any hypothesised turn ‘on’ response must be substantial enough that, despite high field strengths, responsive contrast modulations can still be observed. This might be achieved by maximising the MRI ‘off’ state by combining organic functionalities with responsive paramagnetic architectures to facilitate the greatest possible modulation in water accessibility. It is highly likely that future developments will increasingly consider the integration of ratiometric approaches and so reduce the impact of passive/non-specific [CA] fluctuations. MRET approaches, where concentration-independent measurements are possible by tracking relative *T*_1_- and *T*_2_-weighted signals, against a constant *T*_2_-signature remain particularly interesting. Ratiometric approaches, in general, hold great translational promise,^231^ as they facilitate enhanced diagnostic imaging^232^ by not only bypassing unknown modulations in probe concentration, but also intrinsically self-correcting for spatial and temporal heterogeneity (*e.g.*, within solid tumours)^233^ and measurement artifacts.^234^ This promise is, currently, being held back by synthetic complexity.^235^

It is certainly the case that the clinical utilisation of responsive liposomal MRI CA architectures may benefit from successful liposomal vaccine and drug delivery formulations,^236^ although there remain issues of (in)stability during (long-term) storage and transportation,^237,238^ complexities regarding scale-up,^239,240^ regulatory guidelines,^241^ and targeting/delivery efficiency.^242^ There are also obstacles associated with batch-to-batch consistency.^243^ Microfluidic-assisted synthesis is the most popular technology for generating liposomes with well-controlled size,^244^ and this could indeed potentially enable the large-scale controlled production of liposomal CAs.^245^ Further work will equally have to consider improvements in targeting/localisation through ‘biovectoring’.^246^ An alternative approach to microenvironment specificity might consider logic formulations, whereby AND-/OR-logic gates enable disease-specific imaging and minimised off-target activation, an example of which has been elegantly demonstrated in polymer-coated nanoparticle-based scaffolds.^146^

To conclude, liposomal platforms enable specific size control, payload localisation within the membrane or internal pool, and good circulatory characteristics, all of which are highly tuneable. The finite water pool and limited transmembrane water exchange means that these systems are generally primed for an MRI turn on response, with destructive or non-hydrolytic approaches accessible. The latter include response to a variety of biologically relevant stimuli, such as pH, temperature, redox, and ultrasound. When this is achieved in specific pathophysiological features (such as atherosclerotic plaques,^247^ tumour sites,^248^ ischaemia/stroke^249^) using liposomal scaffolds with diagnostic and therapeutic payloads, this potentially offers clinicians the ability to map disease, providing a continuous feedback loop to further inform patient-specific treatment strategies using MRI.^250,251^

## Author contributions

Anna M. Duncan: original draft preparation, review & editing. Connor M. Ellis: original draft preparation, review & editing. James P. Smith: review & editing. Jason J. Davis: supervision, original draft preparation, review & editing.

## Conflicts of interest

There are no conflicts to declare.

## Data Availability

No primary research results, software or code have been included, and no new data were generated or analysed as part of this review.
